# An injury-responsive *mmp14b* enhancer is required for heart regeneration

**DOI:** 10.1126/sciadv.adh5313

**Published:** 2023-11-29

**Authors:** Ivana Zlatanova, Fei Sun, Roland S. Wu, Xiaoxin Chen, Bryan H. Lau, Pauline Colombier, Tanvi Sinha, Barbara Celona, Shan-Mei Xu, Stefan C. Materna, Guo N. Huang, Brian L. Black

**Affiliations:** ^1^ Cardiovascular Research Institute, University of California, San Francisco, San Francisco, CA 94143, USA.; ^2^ Duke Regeneration Center, Department of Cell Biology, Duke University School of Medicine, Durham, NC 27710, USA.; ^3^ Department of Physiology, University of California, San Francisco, San Francisco, CA 94143, USA.; ^4^ Department of Biochemistry and Biophysics, University of California, San Francisco, San Francisco, CA 94143, USA.

## Abstract

Mammals have limited capacity for heart regeneration, whereas zebrafish have extraordinary regeneration abilities. During zebrafish heart regeneration, endothelial cells promote cardiomyocyte cell cycle reentry and myocardial repair, but the mechanisms responsible for promoting an injury microenvironment conducive to regeneration remain incompletely defined. Here, we identify the matrix metalloproteinase Mmp14b as an essential regulator of heart regeneration. We identify a TEAD-dependent *mmp14b* endothelial enhancer induced by heart injury in zebrafish and mice, and we show that the enhancer is required for regeneration, supporting a role for Hippo signaling upstream of *mmp14b*. Last, we show that MMP-14 function in mice is important for the accumulation of Agrin, an essential regulator of neonatal mouse heart regeneration. These findings reveal mechanisms for extracellular matrix remodeling that promote heart regeneration.

## Teaser

An *mmp14b *endothelial enhancer regulates cardiomyocyte proliferation and regeneration in zebrafish.

## INTRODUCTION

Myocardial infarction remains a leading cause of morbidity and mortality worldwide (*1*, *2*). A central issue is that adult mammals have minimal cardiomyocyte renewal capacity, replacing lost muscle with scar tissue (*2*). In contrast, many organisms, including zebrafish and neonatal mice, have varying degrees of regenerative potential and respond to injury by switching from homeostatic gene expression to a regeneration-specific gene program to modulate essential cellular processes required for regeneration (*3*–*5*). In zebrafish, this includes the profound ability to regenerate the heart following injury through the replacement of lost cardiac myocytes and the resolution of fibrotic scarring (*6*, *7*). Endothelial cells in the heart play key roles in responding to injury and promoting regeneration, and rapid and robust revascularization of the injured area is required for efficient heart regeneration (*8*–*11*).

The extracellular microenvironment is critical for supporting regeneration, and several extracellular matrix molecules are important in promoting heart regeneration, including fibronectin, collagen, and heparin-binding epidermal growth factor–like growth factor (*12*–*15*). Similarly, the large proteoglycan Agrin is enriched in neonatal mouse cardiac extracellular matrix and is required for heart regeneration in neonatal mice (*16*). Moreover, delivery of Agrin to adult mouse hearts at the time of myocardial infarction was shown to increase DNA replication and improve cardiac function after injury (*16*). Important, changes in the extracellular matrix correlate with loss of regenerative potential in mice (*16*–*18*), but the mechanisms through which Agrin and other extracellular matrix molecules are deposited, released, and degraded remain largely unknown.

The matrix metalloproteinase MMP-14 (MT1-MMP) is a membrane-tethered protease implicated in various physiological and pathological processes via modulation of the extracellular matrix, including wound healing, bone development, inflammation, and angiogenesis (*19*). In neuromuscular junctions, MMP-14 is required for the deposition of Agrin (*20*), suggesting a connection between MMP-14 and Agrin-dependent regeneration. Here, we identify MMP-14 as an important positive regulator of heart regeneration. We show that *mmp14b* is required for efficient myocyte proliferation and scar resolution in response to zebrafish heart injury. We also identify an injury-induced, endothelial-specific enhancer from the zebrafish *mmp14b* gene that is required for *mmp14b* induction and for regeneration in response to heart or fin injury. The *mmp14b* endothelial enhancer depends on a conserved TEA domain (TEAD) binding site, implicating the Hippo signaling pathway upstream of *mmp14b*. Moreover, we show that the regulation of *mmp14b* in response to heart injury is conserved in neonatal mice, and pharmacological inhibition of MMP-14 in neonatal mice reduces the presence of Agrin, suggesting a conserved role for MMP-14 in promoting heart regeneration. These studies identify MMP-14 as a crucial, endothelial-expressed regulator of heart regeneration and suggest that strategies to increase MMP-14 expression or activity may positively alter the injury microenvironment to promote myocardial repair following injury.

## RESULTS

### *mmp14b* expression is induced in endothelial cells in response to heart injury in zebrafish

**Fig. 1.**
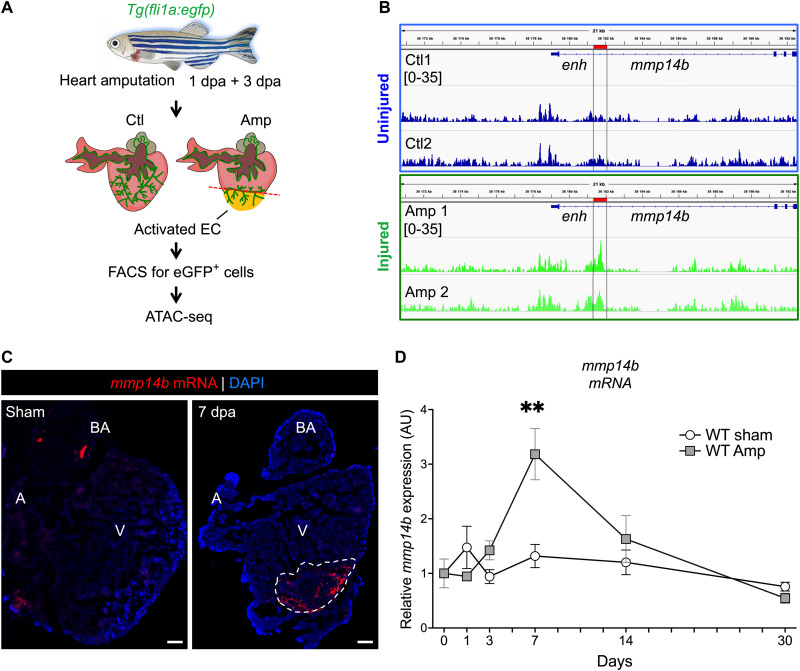
*mmp14b* expression is induced in endothelial cells in response to heart injury in zebrafish. (**A**) Experimental design for heart injury and subsequent ATAC-seq from cardiac ventricle endothelial cells. (**B**) Genome browser view showing ATAC-seq peak (*enh*) at 3 dpa in the *mmp14b* gene in uninjured (Ctl) and injured (Amp) eGFP^+^ cells. (**C**) RNAscope in situ hybridization for *mmp14b* expression in wild-type adult heart sections from uninjured (sham) and injured (7 dpa) hearts. *mmp14b* expression (red fluorescence) is evident in the injured area (dashed line). (**D**) Relative expression of *mmp14b* in wild-type zebrafish hearts expressed in arbitrary units (AU) without injury (sham) in white and injured (Amp) in gray at 1, 3, 7, 14, and 30 dpa as measured by qPCR. DAPI, 4′,6-diamidino-2-phenylindole; A, atrium; V, ventricle; BA, bulbous arteriosus. **P < 0.01.

Given the critical role of endothelial cells in heart regeneration in the zebrafish (*8*–*11*), we sought to identify endothelial genes and gene regulatory regions in the zebrafish that are induced in response to heart injury and regeneration. Therefore, we performed assay for transposase accessible chromatin sequencing (ATAC-seq) on flow-sorted endothelial cells isolated from Tg(*fli1a:egfp*) zebrafish hearts following apical amputation (Fig. 1A). From the ATAC-seq dataset, we examined 1000 chromatin regions (data table S1) that were significantly more accessible (false discovery rate < 0.08; *P* < 0.0004; 2.5-fold increase in accessibility) in endothelial cells isolated from zebrafish hearts at 1 and 3 days post-amputation (dpa) when compared to endothelial cells isolated from uninjured hearts for neighboring genes of interest, particularly those with potential roles in regulating the injury microenvironment. Among the regions that were significantly more accessible in injured compared to uninjured cardiac endothelial cells was a region in the first intron of the *mmp14b* gene (Fig. 1B and data table S1, RegenPk_376). MMP-14 is known to play important roles in angiogenesis and extracellular matrix remodeling in other contexts, and MMPs have been implicated more generally in zebrafish regeneration and are differentially regulated between regenerating and nonregenerating tissue (*19*, *21*–*23*). Therefore, we focused our studies here on Mmp14b as a possible injury-responsive regulator of regeneration in zebrafish.

We reasoned that *mmp14b* expression might be induced in response to injury to promote extracellular microenvironment remodeling and ultimately to promote regeneration. To test this idea directly, we examined the expression of the endogenous *mmp14b* gene by in situ hybridization in response to ventricular resection in adult zebrafish. *mmp14b* transcripts were rare or undetectable in uninjured hearts but were strongly induced in the wound area of injured hearts (Fig. 1C and fig. S1, A and B). Quantification of *mmp14b* expression in sham and amputated ventricles at multiple time points after amputation using reverse transcription quantitative polymerase chain reaction (RT-qPCR) indicated significant induction of *mmp14b* transcripts following injury, peaking at 7 dpa during heart regeneration compared to sham (Fig. 1D). By 30 dpa, *mmp14b* expression in injured hearts had returned to sham levels, indicating a principal role for *mmp14b* during the early phase of cardiac regeneration (Fig. 1D and fig. S1B). We also observed a highly significant, ~6-fold increase in *mmp14b* expression in the caudal fin following amputation (fig. S1C), suggesting that the induction of *mmp14b* is not specific to heart injury but, rather, that it might be generally induced in endothelial cells to promote regeneration in multiple tissues.

### *mmp14b* function is required for efficient heart regeneration

The observation that *mmp14b* was strongly induced in response to heart and fin injury suggested that it might play an important functional role in regeneration. As a first test of this idea, we used a pharmacological strategy to inhibit MMP-14 function. NSC405020 is a highly selective and highly specific inhibitor of MMP-14 collagenase activity (*24*). We first examined the effect of NSC405020 on fin regeneration following amputation over a range of doses (fig. S2, A and B). Treatment with 20 μM NSC405020 significantly inhibited caudal fin regeneration without inducing evident toxicity when compared to dimethyl sulfoxide (DMSO) treatment (fig. S2, A and B). The effect of NSC405020 on caudal fin regeneration was evident as early as 2 dpa (fig. S2, B and C). Inhibition remained evident at 3 and 4 dpa (fig. S2C). We also examined the effect of NSC405020 on heart regeneration following apical ventricular amputation (fig. S2D). We observed a significant decrease in cardiomyocyte proliferation in NSC405020-treated zebrafish compared to controls (fig. S2, D and E). Together, these data demonstrate that inhibition of MMP-14 activity suppresses zebrafish heart and fin regeneration.

**Fig. 2.**
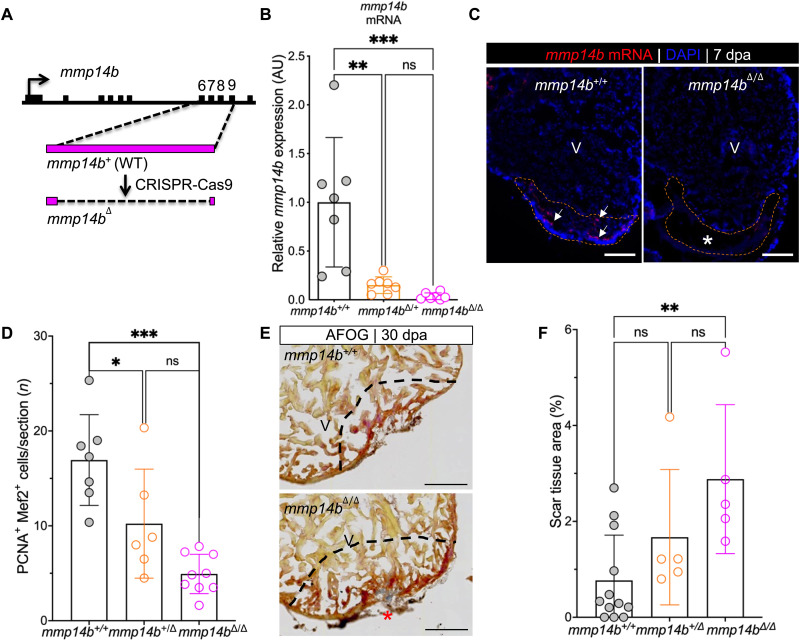
Inactivation of the *mmp14b* gene results in reduced cardiomyocyte proliferation and impaired scar resolution in response to heart injury in zebrafish. (**A**) Strategy used to create an *mmp14b*-null allele. (**B**) Relative *mmp14b* expression in *mmp14b^+/+^*, *mmp14b*^*+/*Δ^, and *mmp14b*^Δ*/*Δ^ larvae as measured by qPCR. (**C**) RNAscope in situ hybridization for *mmp14b* mRNA in *mmp14b^+/+^* and *mmp14b*^Δ*/*Δ^ hearts at 7 dpa. Arrows, *mmp14b* expression in the wild-type *mmp14b^+/+^* heart following injury. The asterisk denotes lack of evident *mmp14b* expression in the *mmp14b*^Δ*/*Δ^ heart. (**D**) Cardiomyocyte proliferation indices at 7 dpa. (**E**) Representative frontal sections of *mmp14b^+/+^* and *mmp14b*^Δ*/*Δ^ hearts collected 30 dpa and stained with Acid Fushin Orange G (AFOG) to detect muscle (brown), fibrin (red), and collagen (blue). The asterisk highlights collagen-rich scar tissue. (**F**) Percent of ventricle composed of scar tissue at 30 dpa in *mmp14b^+/+^*, *mmp14b*^*+/*Δ^, and *mmp14b*^Δ/Δ^ hearts. Data were collected for five to eight sections per heart and averaged to generate each data point. The black dashed lines mark the injured area. Scale bars, 100 μm. Statistical significance was determined using one-way analysis of variance (ANOVA) with Tukey’s multiple comparisons test. ns, not significant; *P < 0.05; **P < 0.01; ***P < 0.001.

As a further test of the requirement of *mmp14b* in heart regeneration, we generated a mutant allele of *mmp14b* using CRISPR-Cas9 (Fig. 2A). This strategy resulted in deletion of 779 base pairs (bp) encompassing exons 6 to 9 of the *mmp14b* gene (Fig. 2A and fig. S3A). *mmp14b* transcripts were essentially undetectable in *mmp14b*^Δ/Δ^ larvae compared to *mmp14b*^+/+^ clutchmates, as shown by RT-qPCR (Fig. 2B). Expression of *mmp14b* was also reduced in *mmp14b*^+/Δ^ heterozygous fish compared to the wild type (Fig. 2B), suggesting gene dosage–dependent expression of *mmp14b*. *mmp14b* expression was undetectable by in situ hybridization on sections of injured adult hearts from *mmp14b*^Δ/Δ^ zebrafish (Fig. 2C and fig. S3B). *mmp14b*-null mutants were viable and fertile, although they were approximately 14% shorter, and some mutant fish exhibited a skeletal defect previously reported in *mmp14a^−/−^*;*mmp14b^−/−^* double mutants (*25*), albeit at low penetrance (one of seven) (fig. S3, C and D).

We compared heart regeneration capacity in *mmp14b^+/+^*, *mmp14b*^*+/*Δ^, and *mmp14b*^Δ/Δ^ following apical amputation. To quantify cardiomyocyte proliferation near the injured area, we costained whole hearts with the cardiomyocyte nuclear marker Mef2 and the cell cycle marker, proliferating cell nuclear antigen (PCNA) (Fig. 2D and fig. S4A). We observed a significant decrease in cardiomyocyte proliferation in both heterozygous and homozygous mutants compared to *mmp14b^+/+^* controls (Fig. 2D). *mmp14b*^Δ/Δ^ fish also had impaired heart regeneration, as evidenced by measurably larger scars at the injury site compared to *mmp14b*^+/+^ clutchmates at 30 dpa (Fig. 2, E and F, and fig. S4B). There are two *mmp14* genes in the zebrafish, *mmp14a* and *mm14b*. We considered the possibility that *mmp14a* might partially compensate for the loss of *mmp14b* in *mmp14b*^Δ/Δ^ zebrafish. However, despite the fact that a partial transcript is likely produced and degraded in *mmp14b*^Δ/Δ^ zebrafish (Fig. 2, A and B), we did not observe an increase in *mmp14a* expression at baseline in the caudal fin of adult *mmp14b*^Δ/Δ^ knockout zebrafish compared to wild type (fig. S3E). Likewise, *mmp14a* transcripts were not induced to a greater level at 7 dpa in hearts of *mmp14b*^Δ/Δ^ knockout zebrafish compared to wild type (fig. S3F). Together, these results demonstrate that *mmp14b* is induced in response to injury, and its function is required in a gene dosage–dependent manner for efficient cardiomyocyte proliferation and heart regeneration.

### An injury-responsive endothelial enhancer resides in the first intron of the *mmp14b* gene

**Fig. 3.**
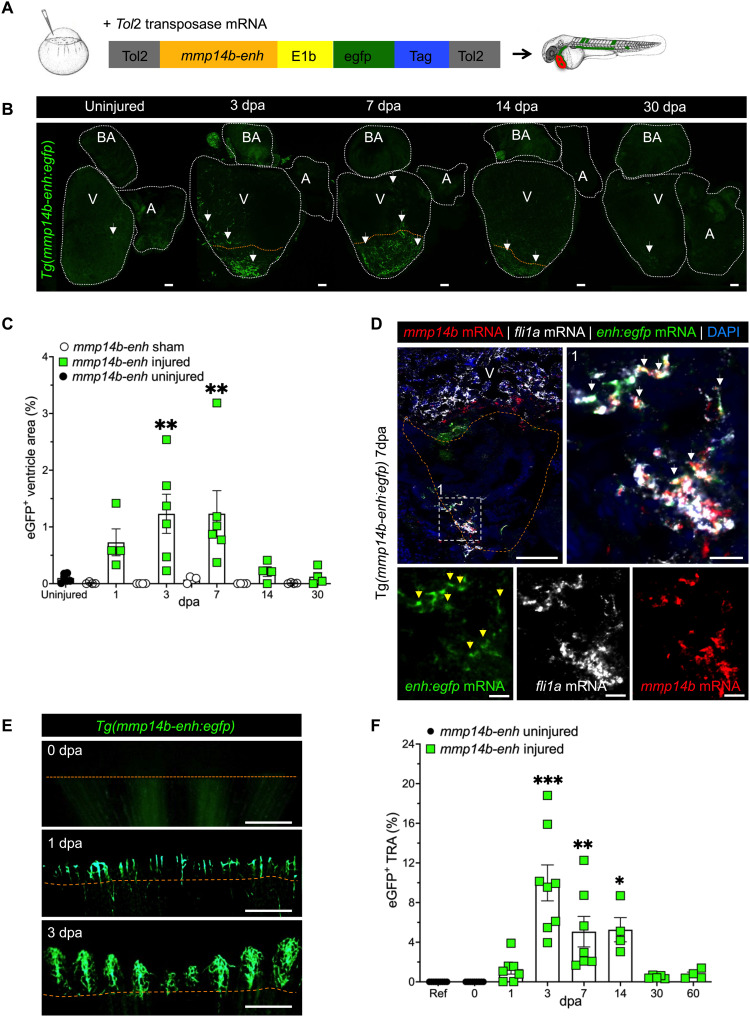
Identification of an injury-responsive *mmp14b* endothelial enhancer. (**A**) Schematic depicting generation of Tg(*mmp14b-enh:egfp*) zebrafish. (**B**) Representative whole-mount views of dissected Tg(*mmp14b-enh:egfp*) zebrafish hearts showing eGFP fluorescence in the vasculature at 3, 7, 14, and 30 dpa, compared to the background fluorescence in the uninjured heart (*n* = 9). White dashed lines outline the heart. Arrows highlight eGFP fluorescence in coronary endothelial cells. (**C**) Quantification of eGFP^+^ cells in Tg(*mmp14b-enh:egfp*) zebrafish in uninjured (black circles), sham-operated (open circles), and amputated (green squares) zebrafish ventricles at 1 to 30 dpa. (**D**) RNAscope in situ hybridization on frontal sections of Tg(*mmp14b-enh:egfp*) hearts at 7 dpa for *mmp14b* (red), *egfp* (green), and the endothelial marker *fli1a* (orange) (white arrowheads). Box 1 (white dashed line) represents the enlarged area to the right and in lower panels, which show individual color channels. (**E**) Fluorescent images of the caudal fin of Tg(*mmp14b-enh:egfp*) zebrafish at 0, 1, and 3 dpa show vascular-specific eGFP expression in newly formed vessels proximal to the amputation plane. (**F**) Vessel density in the total regenerated area (TRA) following caudal fin amputation from 0 to 60 days in Tg(*mmp14b-enh:egfp*) zebrafish (green squares). Uninjured Tg(*mmp14b-enh:egfp*) zebrafish (black circles) are shown as a reference. Orange dashed line approximates the amputation plane. Scale bars, 100 μm [(B) and (D)], 20 μm (D1), and 400 μm (E). Statistical significance was determined using one-way ANOVA with Tukey’s multiple comparisons test (C) or Dunnett’s test (F). *P < 0.05; **P < 0.01; ***P < 0.001.

The identification of *mmp14b* as an injury-responsive gene in endothelial cells stemmed from our ATAC-seq analyses, which defined a region in the first intron of *mmp14b* that became significantly more accessible in endothelial cells following heart injury (Fig. 1B). Since the activation of transcriptional enhancers is strongly correlated with increased transposase accessibility (*26*–*28*) and because enhancers are frequently located in the first intron (*29*, *30*), we hypothesized that the transposase-accessible region of the *mmp14b* first intron might be an enhancer. As an explicit test of this hypothesis, we cloned a 750-bp region surrounding the ATAC-seq peak (*mmp14b-enh*) into an enhanced green fluorescent protein (eGFP) reporter plasmid and generated a stable transgenic zebrafish line, Tg(*mmp14b-enh:egfp*) (Fig. 3A). The *mmp14b-enh* directed robust expression throughout the vascular endothelium during development, except that eGFP expression was notably absent from the developing endocardium (fig. S5, A and B). eGFP expression in adult Tg(*mmp14b-enh:egfp*) was undetectable under normal conditions (Fig. 3B and fig. S5C). In contrast, eGFP expression was strongly induced in response to heart injury (Fig. 3B and fig. S5, C and D). eGFP was detectable as early as 1 dpa with a significant increase in eGFP fluorescence adjacent to and in the wounded area at 3 and 7 dpa compared to sham-operated hearts, which showed minimal detectable eGFP fluorescence (Fig. 3, B and C, and fig. S5, C and D). In situ hybridization showed that *egfp* transcripts expressed from the *mmp14b-enh:egfp* transgene overlapped almost completely with endogenous *mmp14b* transcripts and with transcripts for the endothelial marker *fli1a* (Fig. 3D), indicating that activation of the *mmp14b* enhancer was predominantly restricted to *mmp14b-*expressing endothelial cells. eGFP fluorescence was not detected in cardiomyocytes (fig. S5C).

Consistent with the observed induction of the endogenous *mmp14b* transcript in the fin following amputation, the *mmp14b-enh:egfp* transgene was also rapidly and robustly activated in endothelial cells following caudal fin amputation (Fig. 3E and fig. S5E). Although eGFP was undetectable at baseline (0 dpa), the enhancer was robustly induced by 1 dpa and remained active until at least 14 dpa (Fig. 3, E and F, and fig. S5E). Higher-resolution images of the fin tip showed that eGFP fluorescence in Tg(*mmp14b-enh:egfp*) fish was restricted to a pattern consistent with expression in blood vessels (Fig. 3E). To quantify *mmp14b-enh* activity in response to fin amputation, we measured eGFP^+^ vessel area density (VAD) in the fin following injury (Fig. 3F). Compared to the reference, uninjured fin, we found that *mmp14b-enh*–directed eGFP^+^ fluorescence was significantly up-regulated at 3, 7, and 14 dpa and had returned to baseline expression level by 30 dpa (Fig. 3F). Overall, these results demonstrate that the intronic *mmp14b-enh* is quiescent in adult zebrafish and is robustly reactivated in the endothelium in response to heart and fin injury, and following complete regeneration, its activity is silenced.

### A TEAD motif–dependent core region regulates *mmp14b-enh* during development and in response to injury

**Fig. 4.**
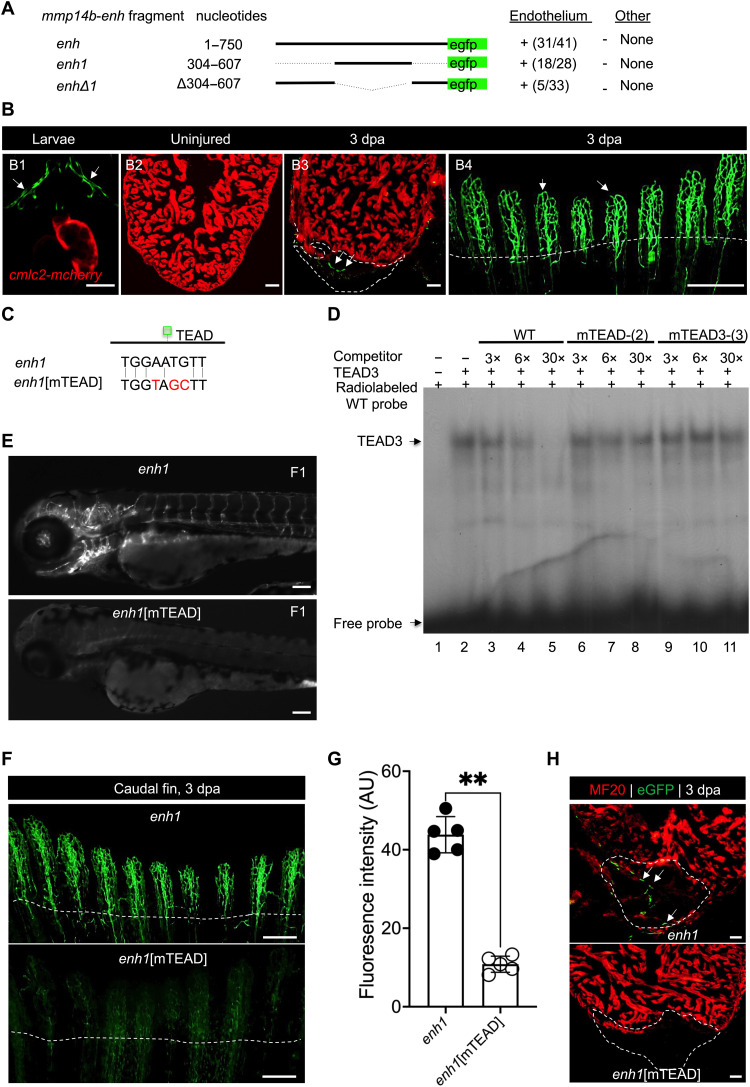
Identification of a TEAD motif–dependent core region of *mmp14b-enh*. (**A**) *mmp14b-enh-egfp* deletion constructs and their activity in transgenic zebrafish larvae at 72 hpf. Activity in endothelial cells and nonendothelial cells (other) is summarized. Numbers represent the fraction of larvae that exhibited activity in the vasculature. (**B**) Representative fluorescent images of Tg(*mmp14b-enh1:egfp*) transgenic zebrafish (B1), uninjured (B2), injured adult hearts (B3), and injured adult caudal fin (B4). eGFP fluorescence (green) indicates activity of the transgene. Cardiomyocytes in [(B1) to (B3)] are marked by *cmlc2:mCherry* (red). (**C**) TEAD site sequences in the *mmp14b-enh1* and *mmp14b-enh1*[mTEAD] transgenes. (**D**) EMSA with recombinant TEAD3 and a radiolabeled, double-stranded *mmp14b-enh1* TEAD site oligonucleotide probe. TEAD3 binding was efficiently competed by unlabeled, self-probe (lanes 3 to 5), but not versions in which two (lanes 6 to 8) or three (lanes 9 to 11) nucleotides were mutated. Unprogrammed reticulocyte lysate control (lane 1). WT, wild type. (**E**) Representative images of F1 generation Tg(*mmp14b-enh1:egfp*) and Tg(*mmp14b-enh1:egfp[mTEAD]*) zebrafish embryos at 72 hpf. (**F**) Representative images of caudal fins from stable transgenic lines, Tg(*mmp14b-enh1:egfp*) and Tg(*mmp14b-enh1:egfp*[mTEAD]) at 3 dpa. eGFP fluorescence (green) indicates activity of the transgenes. (**G**) Quantification of eGFP fluorescence intensity in AU in caudal fins 3 dpa from Tg(*mmp14b-enh1:egfp*) (closed circles) and Tg(*mmp14b-enh1:egfp*[mTEAD]) (open circles) zebrafish. (**H**) Heart sections from stable transgenic lines, Tg(*mmp14b-enh1:egfp*) and Tg(*mmp14b-enh1:egfp*[mTEAD]) at 3 dpa stained with anti-myosin (MF20, red) and anti-eGFP (green) antibodies. Dashed lines mark the amputation planes. White arrows denote endothelial expression. Scale bars, 50 μm [(B1), (B2), (B3), and (H)], 100 μm [(B4) and (E)], and 400 μm (F). Statistical significance in (G) was determined using Student’s *t* test. **P < 0.01.

To define the minimal sequences required for the activity of *mmp14b-enh* in vivo, we tested the ability of various smaller enhancer fragments to direct endothelial-specific activity during development and in response to injury. We found that a smaller enhancer fragment, composed of nucleotides 304 to 607 (*mmp14b-enh1*), directed robust eGFP expression to endothelial cells during development and in regenerating tissues in a manner nearly identical to the full-length *mmp14b-enh* (Fig. 4A). In contrast, deletion of nucleotides 304 to 607 (*mmp14b-enh*Δ*1*) abolished enhancer-driven eGFP expression in vivo (Fig. 4A). Similar to the full-length enhancer, *mmp14b-enh1* directed expression to the vascular endothelium during zebrafish larval development, was quiescent in adult zebrafish, and was reactivated in endothelial cells in adult heart and fin in response to injury and during regeneration (Fig. 4B). Together, these deletion analyses demonstrate that the 304 bp comprising *mmp14b-enh1* are necessary and sufficient to direct endothelial-specific activity during development and regeneration.

To identify cis-regulatory regions responsible for *mmp14b-enh1* function in vivo and to gain insight into the upstream transcription factors regulating the enhancer, we used an unbiased screen for cis-acting elements by using a series of 17 overlapping 21-bp (~2 helical turns) deletions that covered the entire 304 bp of *mmp14b-enh1* (fig. S6A). Each of these deletions was cloned into the eGFP reporter plasmid and analyzed in filial generation 0 (F0) transient transgenic larvae for activity compared to the wild-type *mmp14b-enh1* transgene and to the “empty” transgenic vector, containing only the minimal promoter-*egfp* cassette, for reporter mRNA expression by RT-qPCR (fig. S6A). Using this approach, we found that *mmp14b-enh1*[Δ145-165] had the most significant and largest decrease in enhancer activity compared to the wild-type *mmp14b-enh1* fragment (fig. S6, A and B). The 21-bp deleted region in *mmp14b-enh1*[Δ145-165] encompassed a perfect consensus sequence (TGGAATGTT) for TEAD transcription factor binding (Fig. 4C) (*31*). As expected, given the sequence of the deleted region, it was specifically bound by TEAD3 in electrophoretic mobility shift assays (EMSAs) (Fig. 4D). Mutations of either two or three nucleotides in the core TEAD consensus sequence (Fig. 4C) abolished the specific binding of TEAD3 in EMSA (Fig. 4D), as shown by the failure of the mutant unlabeled probes to compete with the wild-type 21-bp sequence, supporting the notion that the 21-bp region deleted in *mmp14b-enh1*[Δ145-165] contains a bona fide TEAD transcription factor binding site.

To test the function of the TEAD site on *mmp14b-enh1* function in vivo, we mutated the motif from TGGAATGTT to TGGTAGCTT, the same sequence mutation that abolished TEAD3 binding in EMSA, to create *mmp14b-enh1*[mTEAD] and examined the activity of the mutant enhancer in larvae at 72 hours postfertilization (hpf). In F0 transient transgenic analyses, *mmp14b-enh1*[mTEAD] larvae displayed minimal to no eGFP activity; only 2 of 40 transgene-positive larvae showed one or two eGFP-positive cells compared to wild-type *mmp14b-enh1* larvae, where nearly half of all F0 larvae (20 of 38) in the same injection experiment had many eGFP-positive cells. We also generated stable transgenic zebrafish lines, Tg(*mmp14b-enh1*[*mTEAD*]*:egfp*), harboring the mutant TEAD site, which confirmed our F0 analyses: Tg(*mmp14b-enh1:egfp*) displayed robust eGFP fluorescence throughout the developing vasculature, whereas Tg(*mmp14b-enh1*[*mTEAD*]*:egfp*) with the mutant TEAD site had little or no detectable eGFP fluorescence in the developing vasculature (Fig. 4E). Although the TEAD site in *mmp14b-enh1* was required for activity in the developing vascular endothelium (Fig. 4E), it is unlikely that this site alone is sufficient for the endothelial specificity of the enhancer. The 304-bp *mmp14b-enh1* sequence also contains seven perfect consensus binding sites for Ets transcription factors, several members of which are known to play essential roles in endothelial-restricted expression in vivo (*32*, *33*), suggesting that these sites might also contribute to the endothelial activity or specificity of the enhancer.

To determine whether the essential TEAD binding site in *mmp14b-enh1* was required for injury responsiveness of the enhancer, we compared eGFP fluorescence in adult Tg(*mmp14b-enh1*[*mTEAD*]*:egfp*) zebrafish to Tg(*mmp14b-enh1:egfp*) zebrafish in response to fin and ventricle amputation (Fig. 4, F to H). As expected, eGFP expression was robustly activated in adult Tg(*mmp14b-enh1:egfp*) fins following amputation (Fig. 4F). In contrast, mutation of the TEAD site in *mmp14b-enh1* significantly impaired enhancer activity (Fig. 4, F and G). Similarly, wild-type *mmp14b-enh1* was strongly induced 3 dpa in response to apical amputation of the ventricle, while eGFP fluorescence from the *mmp14b-enh1*[mTEAD] transgene was scarcely detectable (Fig. 4H). These results demonstrate that the TEAD binding site in the endothelial-restricted *mmp14b* intronic enhancer is essential for enhancer activation in development and regeneration and suggest an important role for TEAD transcription factors in the injury-dependent activation of *mmp14b*.

### *mmp14b-enh1* is a bona fide *mmp14b* enhancer and is required for efficient heart regeneration

**Fig. 5.**
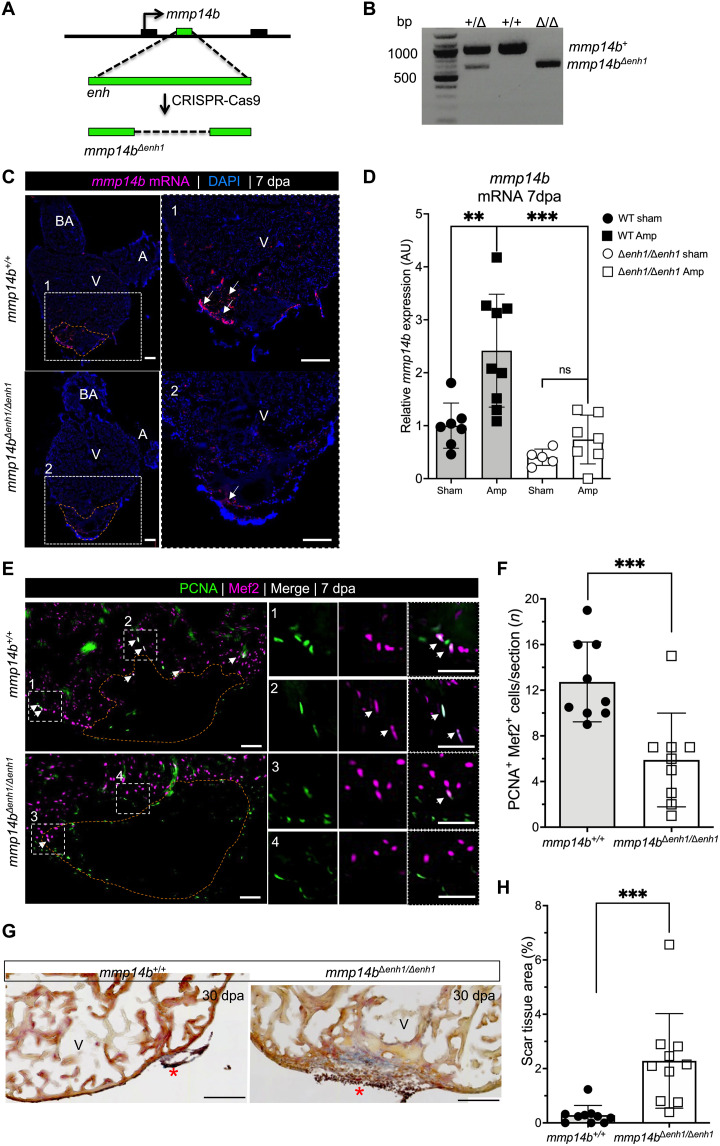
*mmp14b^enh1^* is a bona fide *mmp14b* enhancer and is required for efficient heart regeneration. (**A**) Strategy for deletion of the 304-bp *mmp14b-enh1* sequence. (**B**) PCR genotyping of a *mmp14b*^+/+^, *mmp14b*^+*/*Δ*enh1*^, and *mmp14b*^Δ*enh1/*Δ*enh1*^ zebrafish. (**C**) RNAscope in situ hybridization for endogenous *mmp14b* on frontal sections of injured *mmp14b*^+/+^ and *mmp14b*^Δ*enh1/*Δ*enh1*^ adult zebrafish hearts at 7 dpa. Boxed regions are enlarged in (1) and (2). Arrows highlight *mmp14b* expression (magenta). (**D**) Relative expression in AU of *mmp14b* in control (sham) and injured (Amp) *mmp14b*^+/+^ and *mmp14b*^Δ*ehn/1*Δ*enh1*^ zebrafish hearts 7 dpa. (**E**) Sections of hearts from adult *mmp14b*^+/+^ and *mmp14b*^Δ*enh1/*Δ*enh1*^, subjected to ventricular amputation and immunostained for cardiomyocyte nuclei (⍺-Mef2; magenta) and cycling cells (⍺-PCNA; green). Boxed regions are enlarged in (1 to 4). Arrows highlight PCNA/Mef2 double-positive cardiomyocytes. (**F**) Cardiomyocyte proliferation indices at 7 dpa in *mmp14b*^+/+^ and *mmp14b*^Δ*enh1/*Δ*enh1*^ hearts. Proliferation data were collected for 10 sections per heart and averaged to generate each data point. (**G**) Frontal sections of adult *mmp14b*^+/+^ and *mmp14b*^Δ*enh1/*Δ*enh1*^ hearts 30 dpa stained with AFOG to detect muscle (brown), fibrin (red), and collagen (blue). The asterisk highlights collagen-rich scar tissue. (**H**) Residual scar as a percentage of total ventricular area at 30 dpa in *mmp14b*^+/+^ and *mmp14b*^Δ*enh1/*Δ*enh1*^ hearts; data were collected for four to six sections per heart and averaged to generate each data point. The orange dashed lines approximate the amputation area. Scale bars, 100 μm [(C) and (G)] and 50 μm (E). Statistical significance in (D) was determined using one-way ANOVA with Tukey’s multiple comparisons test. Statistical significance in (F) and (G) was determined using Student’s *t* test. **P < 0.01; ***P < 0.001.

The location of *mmp14b-enh1* in the first intron of the *mmp14b* gene, combined with the similarity in the expression patterns of endogenous *mmp14b* and Tg(*mm14b-enh1:egfp*), strongly suggested that this endothelial-restricted enhancer is a bona fide enhancer of *mmp14b*. As an explicit test of this notion, we used CRISPR-Cas9 to delete the 304 bp that precisely comprises *mmp14b-enh1* from the zebrafish genome (Fig. 5, A and B). We bred *mmp14b*^Δ*enh1*^ zebrafish to homozygosity and then examined *mmp14b* expression in *mmp14b*^Δ*enh1/*Δ*enh1*^ and *mmp14b^+/+^* hearts following apical amputation of the ventricle (Fig. 5, C and D). *mmp14b* was strongly induced and detectable by in situ hybridization on sections of wild-type hearts (Fig. 5C). In contrast, *mmp14b* expression was noticeably reduced in sections from *mmp14b*^Δ*enh1/*Δ*enh1*^ injured hearts (Fig. 5C). RT-qPCR quantification of *mmp14b* mRNA expression showed a statistically significant ~62% reduction in expression 7 dpa in *mmp14b*^Δ*enh1/*Δ*enh1*^ hearts compared to *mmp14b^+/+^* hearts (Fig. 5D). We also observed many fewer *mmp14b* in situ–positive cells in enhancer mutants than in wild-type hearts at 7 dpa (fig. S7A). We observed minimal or no reduction in *mmp14b* expression in *mmp14b*^Δ*enh1/*Δ*enh1*^ larvae compared to wild-type larvae (fig. S7B), indicating that additional *mmp14b* enhancers must also be capable of regulating *mmp14b* expression during development. However, it appears that those same additional enhancers play a minimal role in the reactivation of *mmp14b* in the context of injury and regeneration. These results also suggest that deletion of *mmp14b-enh1* does not result in the inadvertent generation of an *mmp14b*-null allele due to the deletion in the first intron since *mmp14b* expression is unaffected in larval zebrafish. No overt growth phenotype was observed in *mmp14b*^Δ*enh1/*Δ*enh1*^ zebrafish.

We next tested whether *mmp14b-enh1* is required for heart regeneration in zebrafish by comparing cardiomyocyte proliferation and scar resolution in *mmp14b*^Δ*enh1/*Δ*enh1*^ and *mmp14b^+/+^* adults following apical amputation of the ventricle. *mmp14b*^Δ*enh1/*Δ*enh1*^ displayed a significant ~50% reduction in PCNA^+^/Mef2^+^ proliferating myocytes compared to *mmp14b^+/+^* fish at 7 dpa (Fig. 5, E and F). Moreover, we observed impaired scar resolution in *mmp14b*^Δ*enh1/*Δ*enh1*^ enhancer mutants compared to wild-type fish at 30 dpa, with fibrin- and collagen-containing scar tissue significantly more abundant in mutants than in the wild type (Fig. 5, G and H, and fig. S8). Together, these observations demonstrate that *mmp14b-enh1* is a bona fide *mmp14b* enhancer essential for up-regulating *mmp14b* expression in endothelial cells in response to injury and regeneration and that loss of enhancer function results in defective cardiomyocyte proliferation and scar resolution during heart regeneration in zebrafish.

### *Zebrafish mmp14b-enh* is induced by myocardial injury in mice

To determine whether the regulatory networks upstream of the zebrafish *mmp14b-enh* are conserved in mammals, we fused the zebrafish enhancer to the murine *hsp68* promoter and a *lacZ* reporter gene and generated stable transgenic mouse lines. *mmp14b-enh-hsp68::lacZ* displayed vascular endothelial cell–restricted activity during mouse development (fig. S9). As in the developing zebrafish, *mmp14b-enh* activity was absent from the endocardium in the developing mouse embryo (fig. S9, A and B).

**Fig. 6.**
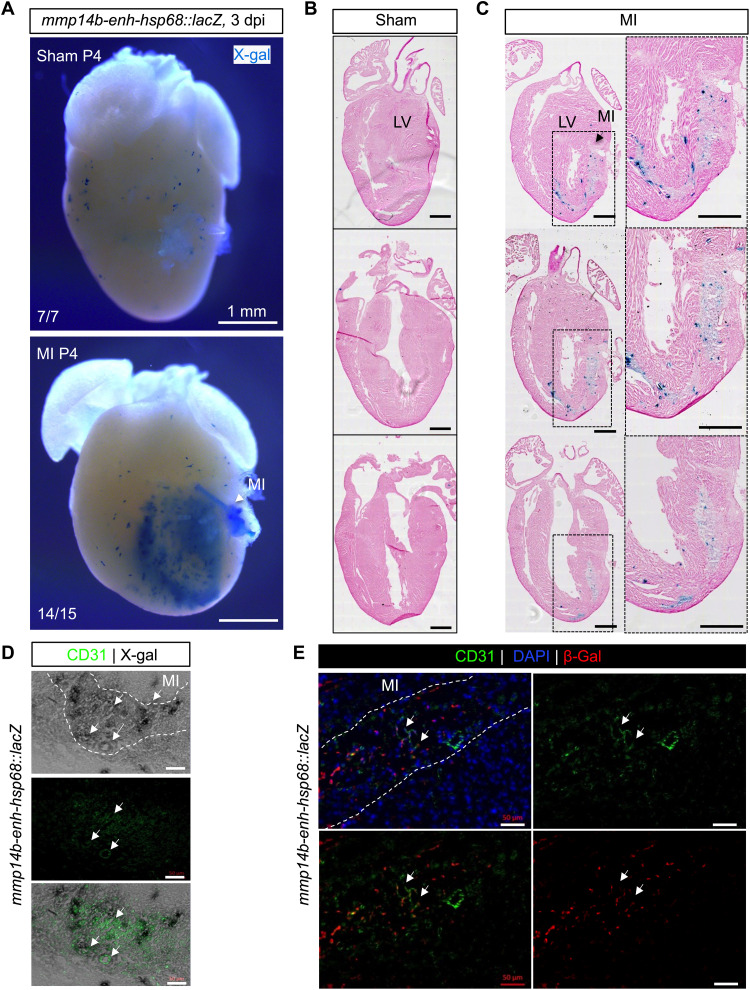
*Zebrafish mmp14b-enh* is induced by myocardial injury in neonatal mice. (**A**) Whole-mount and coronal sections (**B** and **C**) of representative *mmp14b-enh-hsp68::lacZ* hearts in control (sham) and injured myocardial infarction (MI) neonatal mice. Myocardial infarction was induced by permanent coronary ligation in neonatal mice on P1, and β-galactosidase (β-gal) expression was assessed by X-gal staining on P4. Sections were counterstained with hematoxylin and eosin. Note the activation of β-galactosidase (blue staining) in injured *mmp14b-enh-hsp68::lacZ* hearts (14 of 15) but not in sham-operated *mmp14b-enh-hsp68::lacZ* (7 of 7). Boxes in [(C), left] are shown at higher magnification in [(C), right]. LV, left ventricle. (**D** and **E**) Coronal sections of *mmp14b-enh-hsp68::lacZ* neonatal mouse hearts stained for X-gal (gray) (D) or immunostained for β-galactosidase (red) (E) and immunostained for the endothelial cell marker CD31 (green) at 3 dpi. Colocalization of X-gal and CD31 is shown in the bottom of (D). Colocalized expression is highlighted by white arrows. The infarcted area is outlined with a white dashed line. DAPI staining (blue) is shown in (E). Scale bars, 500 μm [(B) and (C)] and 50 μm [(D) and (E)].

Neonatal *mmp14b-enh-hsp68::lacZ* transgenic mice were subjected to myocardial infarction via coronary artery ligation or to a sham operation. Ligation induced robust β-galactosidase activity when compared to sham injury at 3 days post-injury (dpi) (Fig. 6, A to C). X-gal staining in neonatal hearts subjected to coronary ligation appeared to be mostly restricted to CD31^+^ endothelial cells, although some staining in nonendothelial cells was also evident (Fig. 6, D and E). Together, these results demonstrate that the zebrafish endothelial-restricted enhancer, *mmp14b-enh*, is appropriately activated by the mammalian transcriptional machinery during development and in response to injury in mice.

### MMP-14 activity facilitates Agrin availability in the extracellular matrix of neonatal mice

**Fig. 7.**
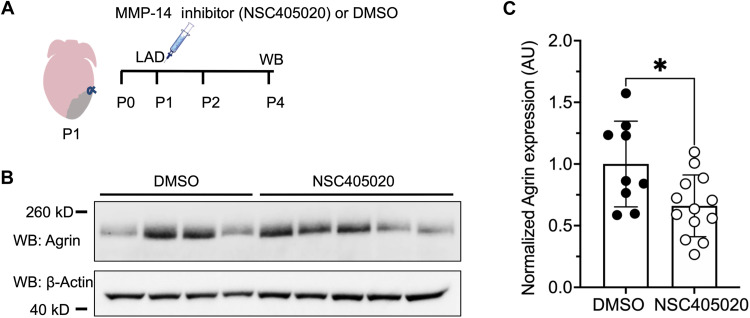
MMP-14 activity facilitates Agrin availability in the extracellular matrix of neonatal mice. (**A**) Schematic of the experimental design for neonatal mouse heart injury and MMP-14 inhibitor treatment. Myocardial injury by coronary artery ligation was conducted on P1, mice were simultaneously treated with a single intraperitoneal injection of 20 μM NSC405020 or DMSO (vehicle control), and hearts were collected on P4 for analysis by Western blot (WB). (**B**) Western blots for Agrin and β-actin from total heart tissue 3 dpi. (**C**) Quantification of the 260-kD isoform of Agrin protein normalized to β-actin and expressed in AU in neonatal mouse hearts following myocardial injury (3 dpi) and treatment with either DMSO or NSC405020. Statistical significance was determined using Student’s *t* test. *P < 0.05.

Recent studies have shown that the large, extracellular matrix proteoglycan molecule Agrin is an essential regulator of neonatal mouse heart regeneration (*16*, *34*), while another recent study has shown that MMP-14 plays a role in Agrin deposition in neuromuscular junctions (*20*). On the basis of these prior observations, we hypothesized that MMP-14 might positively regulate Agrin deposition in the extracellular matrix of neonatal mouse hearts. To test this idea, we induced heart injury in neonatal mice via permanent ligation of the left anterior descending (LAD) artery on postnatal day 1 (P1) and simultaneously treated mice with a single intraperitoneal injection of the MMP-14 inhibitor NSC405020 or DMSO as control (Fig. 7A). NSC405020 caused a significant ~40% decrease in Agrin protein expression on P4 (3 dpi) when compared to DMSO treatment in injured neonatal hearts (Fig. 7, B and C). In contrast, there was no significant difference in Agrin protein or RNA expression in the hearts of uninjured neonatal mice treated with NSC405020 compared to DMSO (fig. S10). Together, these results demonstrate that MMP-14 functions in neonatal mice to regulate the bioavailability of Agrin in the extracellular matrix of the heart following cardiac injury and suggest that MMP-14 may function as an important regulator of heart regeneration in neonatal mice. These results also suggest that the role of MMP-14 as a positive effector of heart regeneration via proteolytic activity in the extracellular matrix is conserved between fish and mammals.

## DISCUSSION

### A metalloproteinase-dependent program promotes heart regeneration via regulation of the injury microenvironment

Creating a microenvironment that is conducive to regeneration requires the coordinated actions of multiple cell types and the activation of numerous proregenerative processes, including the formation of new blood vessels, scar resolution, and growth factor and cytokine signaling that lead to cell cycle reentry (*12*, *13*). Here, we found that the membrane-tethered MMP MMP-14 is induced in response to heart injury and facilitates regeneration in the zebrafish. It is likely that MMP-14 works by multiple mechanisms to promote regeneration. MMP-14 is a collagenase that directly degrades scar tissue (*35*). In addition, MMP-14 is essential for endothelial cell migration, invasion, and assembly into tube networks to form new vasculature (*21*). MMP-14 is known to regulate angiogenesis in several contexts (*21*, *35*, *36*), suggesting that at least part of the proregenerative role of MMP-14 observed here might be due to its role in promoting angiogenesis. MMP-14 is primarily expressed in the sprouting tip of vascular endothelial cells, a site where cell proliferation and focal degradation of collagen preferentially occur (*21*). The idea that MMP-14 promotes regeneration via its proangiogenic functions is particularly interesting given the critical proregenerative role played by the coronary vasculature during heart regeneration (*8*–*10*). Our observation that *mmp14b* is transiently induced via an endothelial-restricted enhancer in response to heart injury is consistent with the critical proregenerative roles of the coronary vasculature. Notably, MMP-14 is also expressed in cell types other than endothelium, including macrophages (*37*), a cell type that one might expect to be involved in the injury response and clearance of debris away from the injured area (*38*, *39*). Although we cannot rule out a proregenerative role for MMP-14 produced by other cell types, our work here shows that *mmp14b* is predominantly induced in endothelial cells in response to heart injury and that endothelial expression of *mmp14b* is essential for efficient heart regeneration.

### MMP-14 functions as an upstream and downstream effector of Hippo signaling

The Hippo signaling pathway functions as an important inhibitor of cell proliferation, and Hippo pathway suppression is crucial for heart regeneration (*12*). When Hippo signaling is off, the transcriptional coactivator proteins Yes-associated protein (YAP) and transcriptional coactivator with PDZ-binding motif (TAZ) translocate to the nucleus, where they activate transcription via interaction with DNA binding transcription factors, most notably members of the TEAD family, to promote cellular proliferation (*40*, *41*). In adult mice, expression of constitutively active YAP leads to increased cardiomyocyte renewal and improved contractility after myocardial infarction (*42*, *43*). Here, we show that the *mmp14b* enhancer contains a perfect consensus TEAD binding site, which is bound by TEAD3 and is required for enhancer activation in response to injury (Fig. 4). While *mmp14b-enh* is rapidly induced following injury and remains active during most of the tissue repair process in zebrafish, its activity extinguishes as the injury is resolved (Fig. 3). Although the mechanism through which the *mmp14b* enhancer returns to the quiescent state is unknown, it is interesting to speculate that this might occur because of the restoration of Hippo signaling and silencing of TEAD-dependent activation of the enhancer (*44*). Alternatively, restoration of normal contraction or extracellular matrix stiffness might influence YAP nuclear export, resulting in enhancer silencing via the TEAD site in the *mmp14b* enhancer (*45*, *46*). Overall, our data support the idea that *mmp14b* is a target of “Hippo off” and that MMP-14 and its proteolytic activity may serve as a downstream effector of the pathway via extracellular matrix remodeling.

MMP-14 may also function upstream of Hippo signaling via its positive effect on the availability of the proregenerative molecule Agrin (*20*). Previous work has shown that Agrin interacts with Dystroglycan 1, a component of the dystrophin-glycoprotein complex, in murine cardiomyocytes to activate extracellular signal–regulated kinase (ERK) signaling and also to induce YAP/TAZ nuclear translocation (*16*). Our work supports the idea that MMP-14 produced by endothelial cells increases Agrin availability in the extracellular microenvironment (Fig. 7, B and C), potentially allowing it to bind to the dystrophin-glycoprotein complex to inhibit Hippo signaling in cardiomyocytes and thus promote their cell cycle reentry (*42*, *47*).

### MMP-14 is a potential therapeutic target for heart regeneration and repair

Promoting endogenous heart repair in humans is an important challenge in regenerative medicine (*6*, *48*). The work presented here suggests that MMP-14 might be useful as a therapeutic target for heart regeneration in multiple ways. We show that the endogenous zebrafish *mmp14b* gene is induced transcriptionally in endothelial cells in response to heart injury and that its function is important for efficient heart regeneration. The mouse *Mmp14* gene is also induced in endothelial cells in response to heart injury. In a recent study, Quaife-Ryan and colleagues (*49*) performed RNA sequencing on isolated cell populations from adult mouse hearts following myocardial infarction and in sham-operated controls. We analyzed their published datasets and found that *Mmp14* expression was induced 2.98-fold (*n* = 4 per group, *P* < 0.001) in endothelial cells in hearts subjected to myocardial infarction compared to sham controls (*49*). These results indicate that *Mmp14* is induced in endothelial cells in mice in response to cardiac injury and suggest that strategies to further augment the endogenous expression of *Mmp14* might have therapeutic benefit in mammals.

The *mmp14b* enhancer described in these studies might also serve as a valuable therapeutic tool for regeneration. The enhancer functions as an endothelial-restricted, injury-responsive enhancer, and as noted above, its activity returns to quiescence upon injury resolution (Fig. 3). These features of the *mmp14b* enhancer: (i) responding to injury, (ii) remaining active for all or most of the period of regenerative healing, and (iii) returning to quiescence upon injury resolution meet the criteria for a “regeneration enhancer” or tissue regeneration enhancer element (TREE), which is a more narrowly defined subtype of injury-responsive enhancer (*50*). These regeneration enhancer properties make the zebrafish *mmp14b* enhancer an interesting candidate enhancer to deploy proregenerative molecules in response to injury, taking advantage of the enhancer’s return to quiescence following injury resolution. We recently used this type of strategy using a zebrafish *lepb*-linked regeneration enhancer to direct expression of proregenerative molecule YAP5SA to the mammalian heart using engineered adeno-associated virus vectors, resulting in increased myocyte proliferation and improved heart function without the complication of continuous, unregulated delivery and associated pathological effects of uncontrolled proliferation (*51*). Because the *mmp14b* enhancer described here is an endothelial-restricted regeneration enhancer, its potential to deploy proregenerative cargoes is not restricted to the heart. Instead, *mmp14b-enh* could potentially be used to promote regeneration in any vascularized tissue.

Other strategies to promote adult mammalian heart regeneration involve manipulating the injury microenvironment to promote cardiomyocyte proliferation and reduce scarring via alteration of the extracellular matrix (*52*, *53*). In this regard, it is important to note that MMP-14 is an extracellular, membrane–bound metalloproteinase with direct collagenase activity (*35*). Our work here shows that its activity can increase the availability of Agrin in the extracellular matrix of neonatal mice (Fig. 7, B and C). Thus, approaches to up-regulate the activity or function of MMP-14 or via inhibition of tissue inhibitor of metalloproteinase activity, manipulation of microRNAs, or other strategies might facilitate regenerative repair in a manner similar to what we have shown here.

## MATERIALS AND METHODS

### Zebrafish models

The outbred wild-type zebrafish (*Danio rerio*) Ekkwill (EKW) strain, Tg(*fli1a:egfp*)^y1^, Tg(*cmlc2:mCherry*)^s890^, and Tg*(flk1:ras-cherry*)^s896^ have been described previously (*54*–*57*). To generate the Tg(*mmp14b-enh:egfp*)^sfc26^ zebrafish line, a 750-nucleotide fragment of the *mmp14b* intron, spanning chromosome 2:38181326-38182075, was cloned by PCR from zebrafish genomic DNA using the following primers: enhF, 5′-ctccatatgggctcttttcttcctctgtttc-3′ and enhR, 5′-aaaacatttctaattgcaggtcaatgactactagtttc-3′. The resulting fragment was cloned into the Tol2 transgenic vector E1b-GFP-Tol2 (*58*) (RRID: Addgene_37845). Tg(*mmp14b-enh1:egfp*)^sfc27^, Tg(*mmp14b-enh*Δ*1:egfp*), and Tg(*mmp14b-enh1*[mTEAD]:*egfp*)^sfc28^ zebrafish lines were generated from Tol2 constructs with the appropriate mutation or deletion in the *mmp14b-enh* sequence generated by PCR from the 750-bp *mmp14b-enh* sequence in the Tol2 plasmid (E1b-GFP-Tol2) (*58*) (RRID: Addgene_37845). All plasmid sequences were confirmed by Sanger sequencing before zebrafish injection. Twenty-five nanograms of E1b-Tol2-eGFP plasmid containing the fragments and 125 ng of Tol2 mRNA were injected into wild-type and Tg(*cmlc2:mCherry-NTR*)^s890^ zebrafish embryos at the one-cell stage after purification, as previously described (*59*). Oligonucleotides used for enhancer amplification are listed in table S1. F0 founder embryos were raised to 72 hpf before imaging, and enhancer activity was scored (average *n* = 120). F0 embryos injected with individual enhancers were raised to generate stable transgene lines.

Mutant zebrafish were generated by CRISPR-Cas9 genome editing, as previously described (*60*), using Alt-R S.p. HiFi Cas9 Nuclease V3 (Integrated DNA Technologies). Single-guide RNAs (sgRNAs), 5′-gagactaacagagaaatgaa**agg**-3′ and 5′-tggctagtcacatccttcat**agg**-3′, targeting genomic sequence 5′ and 3′ of the *mmp14b-enh1* were designed using CRISPR ID (*61*). Fifty picograms of individual sgRNAs and 150 pg of Cas9 protein were mixed in a final volume of 2.5 μl, incubated at 37°C for 5 min, and then 0.5 to 1 nl of the mixture was injected into one-cell stage embryos in either the yolk or the cell cytoplasm, as indicated. A total of 0.05% phenol red dye was added for visualization of injections.

F0 zebrafish were genotyped by PCR and/or DNA sequencing from genomic DNA extracted from tail clips using standard procedures, and further mating of F0 zebrafish was performed to generate F1 heterozygotes, F2 heterozygotes, and F3 homozygotes. Embryos were imaged and then genotyped by PCR analysis of genomic DNA isolated from whole embryos. The *mmp14b*^Δ*enh1*^ mutant allele carries a 304-bp deletion in the *mmp14b* intron, as depicted in Fig. 5. An intercross of *mmp14b*^*+/*Δ*enh1*^ heterozygous fish did not produce any evident phenotype at 72 hpf. To generate *mmp14b* knockout fish, the following sgRNAs were used: 5′-ggagtgtgatggggcgtctc**agg**-3′ and 5′-gcattcatgagtcgagatca**agg**-3′. A schematic of the *mmp14b* mutant allele is illustrated in Fig. 2. Primer sequences used for genotyping are shown in table S1.

### Transgenic mouse models

The outbred wild-type mouse (*Mus musculus*) strain CD-1 was used. Transgenic mice were generated by oocyte microinjection as described previously (*62*). To generate the transgene construct, a fragment from zebrafish genomic DNA corresponding to the 750-bp zebrafish *mmp14b* enhancer sequence described above was subcloned into the transgenic reporter plasmid *hsp68-lacZ* (*63*). The transgene was released by digestion with appropriate restriction enzymes, purified using the Qiagen gel extraction kit (catalog no. 28,704) and diluted to 2 ng/µl before injection. Transgenic founders were either collected for transient transgenic analyses or used to establish stable transgenic lines. F0 founder mice were genotyped using the MyTaq Extract-PCR Kit (Bioline catalog no. BIO-21126).

### Animal care

The University of California, San Francisco Institutional Animal Care and Use Committee approved all animal studies. Experiments were performed in accordance with the Public Health Service Policy on the Humane Care and Use of Laboratory Animals. Results are reported in concordance with the ARRIVE guidelines (*64*).

### Adult zebrafish heart injury

Adult zebrafish between 4 and 8 months old were anesthetized by immersion into 0.04% tricaine (Sigma-Aldrich) and immobilized on a foam holder mounted on a sponge. Partial ventricular resection surgeries were performed in which ~20% of the cardiac ventricle was removed at the apex. For analysis of regeneration, animals were euthanized at different times after amputation by immersion in 0.016% tricaine, and hearts were dissected. To assess the amputation area caused by the procedure, photographs of resected hearts were taken between 1 and 60 dpa. The damaged area was identified from the accumulation of blood at the injury site and quantified by analysis of histological sections as previously described (*7*).

### Adult zebrafish fin amputation, inhibitor treatment, and regeneration assessment

Adult zebrafish were anesthetized in 0.04% tricaine in system water, and caudal fins were amputated by using a scalpel. The fish were allowed to recover in fresh system water, and fins were allowed to regenerate at 28°C until the indicated time points. Caudal fin tissue, two bone segments proximal to the amputation plane, was collected for gene expression analyses. For inhibitor studies, following amputation, zebrafish were divided into 500-ml tanks, the MT1-MMP inhibitor NSC405020 (Sigma-Aldrich) was added in DMSO at a final inhibitor concentration of 20 μM, and caudal fin regeneration in the presence of inhibitor, compared to the addition of 0.1% DMSO alone, was assessed at the indicated time points. Fish were maintained at 28°C, and water and inhibitor were refreshed daily. At 4 dpa, fish were removed from the tanks, anesthetized, photographed, and euthanized. Tissue regrowth and vessel regeneration were measured for each individual animal, and data were expressed as a percentage of vascularization, referred to as VAD, as previously described (*65*). Briefly, the total regenerated area (TRA), regardless of vascularization, was measured. The vascular projection area (VPA), which is the area of all vessels within the regenerated area as projected on fluorescence images, was also measured. VAD represents the ratio of the VPA to TRA.

### Mouse embryo collection and X-gal staining

The day of the vaginal plug was designated as embryonic day 0.5 (E0.5), and embryos were collected at E10.5 and processed appropriately for X-gal staining and immunostaining. Section and whole-mount X-gal staining to detect β-galactosidase activity and counterstaining with nuclear fast red were performed with murine tissue as described previously (*66*).

### Myocardial injury in neonatal and adult mice

Myocardial injury was performed in wild-type CD-1 and in transgenic *mmp14b-enh-hsp68::lacZ* male and female P1 neonatal mice by coronary ligation of the LAD under anesthesia, similarly to previously described methods (*67*). Following ligation, hearts were collected on P4 for histology and Western blot analysis. Adult (8-week-old) transgenic *mmp14b-enh1-hsp68::lacZ* male and female mice were subjected to myocardial infarction by ligating the LAD under anesthesia, as previously described with minor modifications (*68*). Thoracotomy was performed to the left of the sternum to expose the heart followed by opening of the pericardium and placement of a 7-0 Prolene suture beneath the LAD. Coronary artery ligation was confirmed by observing blanching of the distal circulation at the ventricular apex. At 3 dpi, hearts were collected and processed for X-gal staining as described (*66*).

### ATAC-seq and bioinformatic analysis

ATAC-seq experiments were performed on uninjured and injured Tg(*fli1a:egfp*)^y1^ adult zebrafish hearts. One and 3 dpa ventricles were collected and pooled, dissociated with Liberase DH (Roche 5401054001) at 37°C, and centrifuged at 1250 rpm for 8 min at 4°C. Cells were resuspended in 1.5 ml of Dulbecco’s modified Eagle’s medium and passed through a 70-μm filter. GFP-positive cells were fluorescence-activated cell–sorted and collected in tubes and pelleted, and then the total DNA was isolated for ATAC-seq as previously described (*26*). Sequencing was performed using Illumina HiSeq 2000, and sequences were aligned to GRCz10, as previously described (*69*). Local peaks were identified from sequencing data using MACS2 callpeak (--nomodel --shift -37 --extsize 73 -q 0.05) (*70*). A set of peak coordinates common to all sequencing datasets was generated using the bedops merge function (*71*). Sequencing reads were mapped to these peaks using subread featurecounts (*72*). Differential read mapping between uninjured and injured fli1a^+^ cells was calculated using edgeR to identify peaks with differential accessibility (*73*).

### RNA extraction, cDNA synthesis, and qPCR

Total RNA was isolated from uninjured and injured adult hearts using TRIzol (Thermo Fisher Scientific), according to the manufacturer’s instructions. Five to 10 male and female adult zebrafish hearts were collected for each condition. For qPCR from zebrafish larvae, 15 larvae at 72 hpf were pooled for each condition, and total RNA was isolated using the RNeasy Micro kit (Qiagen), according to manufacturer’s instructions. In each case, 100 ng of total RNA was reverse-transcribed to cDNA with the QuantiTect Reverse Transcription Kit (Qiagen), and real-time qPCR was performed using SYBR Green (Applied Biosciences) in an iQ5 PCR system (Bio-Rad). Primer set efficiency was calculated and adjusted using LinRegPCR (*74*). Detection of the *ef1a* housekeeping gene was used to normalize gene expression in the qPCR experiments. Experiments were done in triplicate. The primers used for qPCR are listed in table S1.

### Immunofluorescence, histochemistry, and RNAscope in situ hybridization

Adult zebrafish hearts were dissected and fixed in 4% paraformaldehyde in phosphate-buffered saline (PBS) (pH 7.4) for 1 hour at room temperature on a nutator. Hearts were then washed three times in a fish-fix buffer containing 3% sucrose, 1 M CaCl_2_, 0.2 M Na_2_HPO_4_, and 0.2 M NaH_2_PO_4_, as described previously (*75*). The tissues were cryopreserved overnight at 4°C in 30% (w/v) sucrose solution prepared in 1× PBS. The hearts were then embedded in OCT (optimal cutting temperature) compound (Tissue-Tek) and stored at −80°C. For Acid Fushin Orange G (AFOG) staining, cryosections were fixed with Bouin’s solution for 1 hour at 56°C and stained with hematoxylin solution, according to the manufacturer’s instructions (Diapath, S.p.A., Salvodini, Italy). For immunofluorescence, OCT was removed from 8-μm-thick cryosections by rinsing the slides with 1× PBS. The sections were then permeabilized with 0.5% Triton X-100 in 1× PBS for 30 min at room temperature, followed by incubation in blocking buffer (5% bovine serum albumin, 10% goat serum, 0.3% Tween-20, 0.2% Triton X-100, and 1% DMSO). Later, the sections were incubated with primary antibodies overnight at 4°C under sealed coverslips. After washing with 1× PBS for 15 min, sections were incubated with secondary antibodies for 1 hour at room temperature and washed with 1× PBS for 15 min, nuclei were stained with a 1:10,000 dilution of 4′,6-diamidino-2-phenylindole (DAPI), and slides were mounted in Mowiol mounting medium (Sigma-Aldrich, catalog no. 81381). Primary antibodies used for immunofluorescence are listed in table S2. The following antibody dilutions were used: anti-myosin heavy chain (MF20), 1:50; anti-PCNA, 1:200; anti-Mef2, 1:50; anti-GFP, 1:200; anti–β-galactosidase, 1:200; anti-CD31, 1:50; and Alexa Fluor–conjugated secondary antibodies, 1:200.

For RNAscope, zebrafish hearts were rapidly dissected, immediately embedded in OCT, and frozen on dry ice. Blocks were sectioned into 8-μm-thick sagittal slices onto positively charged slides using a Leica CM3050 S Research Cryostat. Slides were dried in the cryostat and then stored at −80°C for up to 2 weeks. RNAscope in situ hybridization was performed according to the manufacturer’s instructions for fresh frozen sections [Advanced Cell Diagnostics (ACD)]. ACD designed customized color channel–specific DNA oligonucleotide probes for *mmp14b*, *fli1a*, and *egfp*. Each probe was tested in at least 10 zebrafish. RNA-scope probes are listed in table S2. Probe targets were visualized using Opal dyes 520, 570, 620, or 690 (Akoya), listed in table S1. Slides were mounted with ProLong Gold Antifade Mountant (Life Technologies) or VECTASHIELD Antifade Mounting Medium (Vector Laboratories).

Fluorescent and bright-field images were acquired using either a Leica MZ165 FC stereomicroscope equipped with a DFC450 camera using the Leica Application Suite software package or a Zeiss LSM 700 confocal microscope or Zeiss Axioscan 4 microscope with Zeiss ZEN software (blue edition) used for image settings. The Fiji software package was used to generate maximum intensity projections and adjust brightness and contrast.

### Western blot

Total heart tissue extracts were prepared with radioimmunoprecipitation assay buffer with a fresh protease inhibitor cocktail, as described previously (*76*). Proteins were separated by SDS–polyacrylamide gel electrophoresis and transferred to Imobilon polyvinylidene difluoride membranes (Invitrogen, catalog no. B24002) using standard procedures. Following transfer to the membranes, blots were incubated in blocking solution [5% skim milk in tris-buffered saline with 1% Tween-20 (TBST)] with agitation for 1 hour at room temperature and then incubated with primary and secondary antibodies, and chemiluminescence detection was conducted as previously described (*76*). The antibodies used are listed in table S2. Anti-Agrin was used at a 1:1000 dilution. Anti–β-actin was used at a 1:1000 dilution. Horseradish peroxidase–conjugated anti-mouse or anti-rabbit secondary antibody was used at a 1:5000 dilution.

### Electrophoretic mobility shift assays

EMSA experiments were performed as previously described (*62*). TEAD3 protein was synthesized in vitro from pCITE-2a (Novagen, catalog no. B050) expression plasmids using T7 polymerase using the TNT Quick Coupled Transcription/Translation System (Promega), according to the manufacturer’s instructions. The oligonucleotide probe sequences used for EMSA are listed in table S1.

### Statistics and reproducibility

A minimum of five independent biological replicates were performed for each experiment. Statistics and graphical representations were performed using Prism 9.2 (GraphPad Software Inc.). Quantitative results are reported as the mean ± SD. *n* values and group sizes for individual experiments are presented in the graphs as individual points or are indicated in the figure legends. Details for statistical tests for each experiment are indicated in the figure legends.
