# Supplementary material for: An injury-responsive mmp14b enhancer is required for heart regeneration
Source: Sci Adv. 2023 Nov 29;9(48):eadh5313. doi: 10.1126/sciadv.adh5313 (PMC10686572; doi:10.1126/sciadv.adh5313)
Supplement: Supplementary file 1 — Figs. S1 to S10 Table S1 and S2 Legend for data S1 [file sciadv.adh5313_sm.pdf]

Science Advances
AAAS

Supplementary Materials for

An injury-responsive *mmp14b* enhancer is required for heart regeneration

Ivana Zlatanova *et al.*

Corresponding author: Brian L. Black, brian.black@ucsf.edu

Sci. Adv. **9**, eadh5313 (2023)
DOI: 10.1126/sciadv.adh5313

The PDF file includes:

Figs. S1 to S10
Tables S1 and S2
Legend for data S1

Other Supplementary Material for this manuscript includes the following:

Data S1

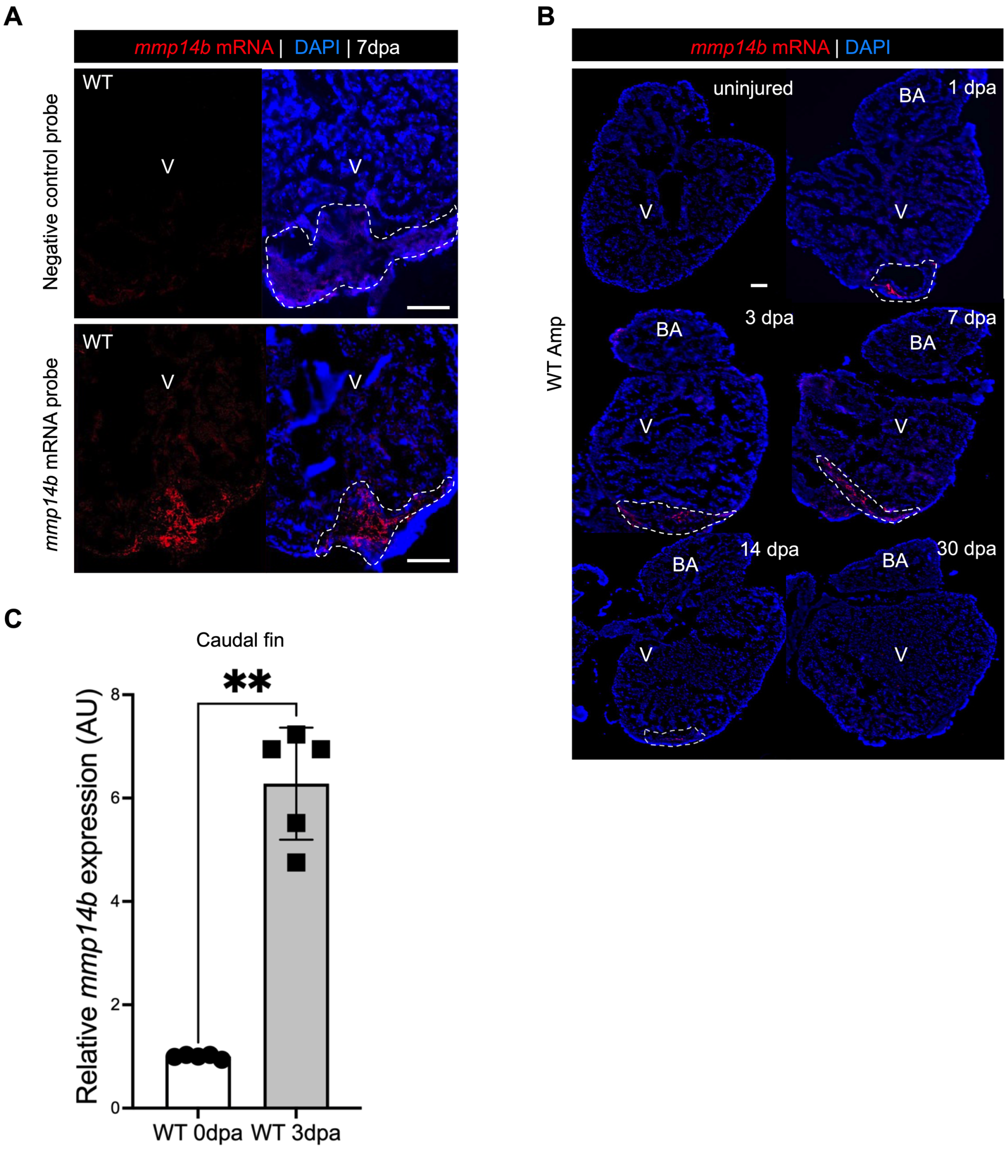

Fig. S1. *mmp14b* transcript expression during heart regeneration. (**A**) RNAscope *in situ* hybridization on sections of wild type zebrafish hearts at 7 dpa using an *mmp14b*-specific probe and negative control probe. (**B**) RNA-scope *in situ* hybridization for *mmp14b* expression in wild type zebrafish hearts at 1, 3, 7, 14, and 30 dpa. 6–8 sections from 6 hearts were analyzed. (**C**) Relative expression of *mmp14b* in wild type zebrafish caudal fin without injury and injured at 3 dpa as measured by RT-qPCR. Scale bars, 100 µm (A and B) Statistical significance was determined using student's t-test. **, $p<0.01$.

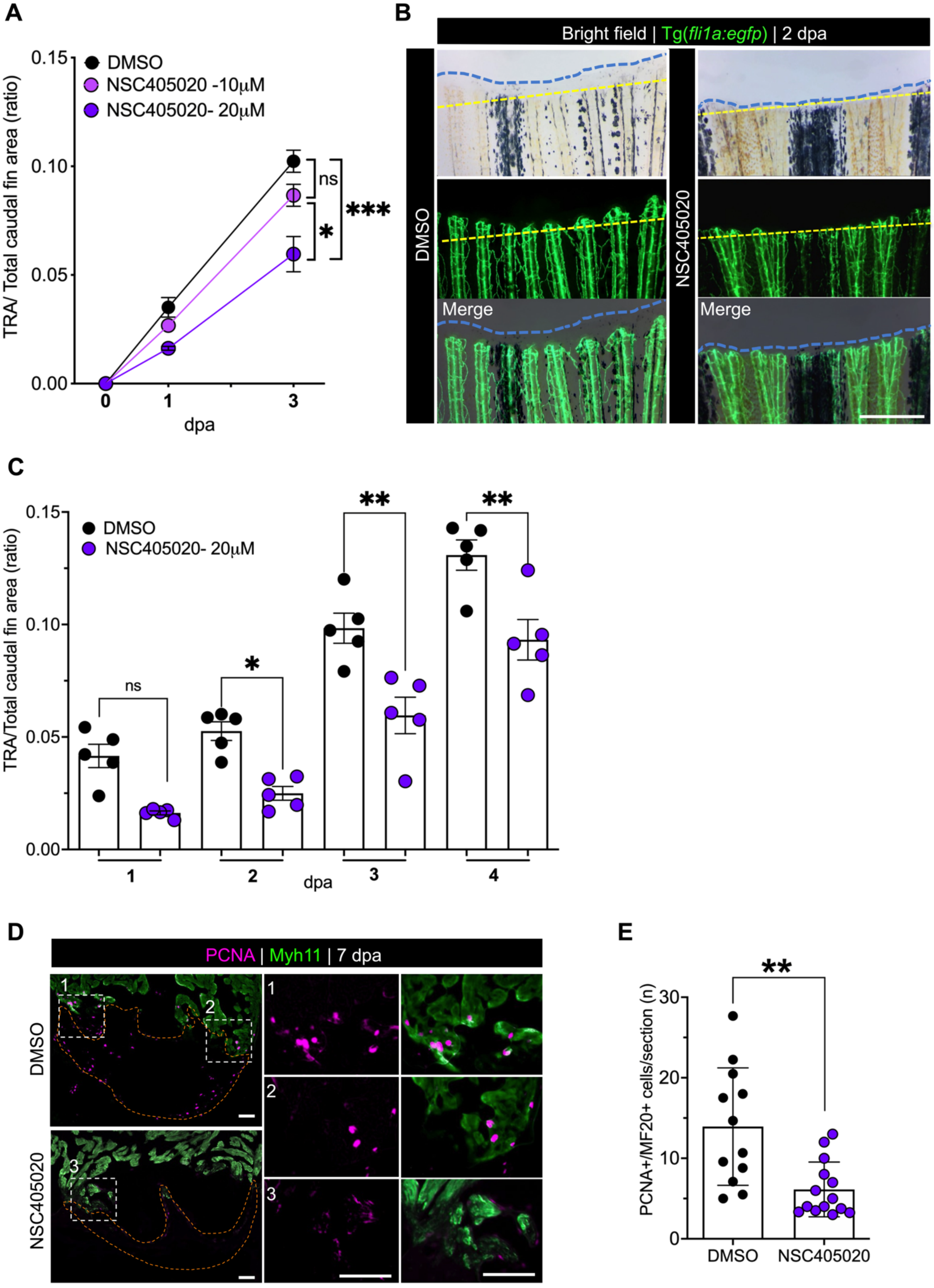
A
DMSO
NSC405020 -10μM
NSC405020- 20μM
TRA/ Total caudal fin area (ratio)
0.15
0.10
0.05
0.00
ns
*
***
0
1
3
dpa
B
Bright field | Tg(fli1a:egfp) | 2 dpa
DMSO
NSC405020
Merge
Merge
C
DMSO
NSC405020- 20μM
TRA/Total caudal fin area (ratio)
0.15
0.10
0.05
0.00
ns
*
**
**
1
2
3
4
dpa
D
PCNA | Myh11 | 7 dpa
DMSO
NSC405020
1
2
3
E
**
PCNA+/MF20+ cells/section (n)
30
20
10
0
DMSO
NSC405020

Fig. S2. Pharmacologic inhibition of Mmp14b function impairs regeneration in zebrafish. (**A**) Assessment of two concentrations of the MMMP-14 inhibitor NSC405020 on caudal fin regeneration. Tissue regeneration was significantly inhibited at 3 dpa in the presence of 20 µM but not in the presence of 10 µM NSC405020 when compared to DMSO; n=5-8 fish per group. (**B**) Representative bright field and fluorescent images of Tg(*fli1a:egfp*) zebrafish subjected to caudal fin amputation and either DMSO or 20 µM NSC405020 at 2 dpa. Dashed blue lines approximate the blastema progression; dashed yellow lines represent the injury site. Note the inhibition of vessel growth and blastema progression in the presence of NSC405020 compared to DMSO. (**C**) Quantitative assessment of the total regenerated area (TRA) following caudal fin amputation in wild type zebrafish at 1, 2, 3, and 4 dpa in the presence of DMSO and 20 µM NSC405020. (**D**) Heart sections from DMSO- and NSC405020-treated zebrafish subjected to ventricular amputation. Heart sections at 7 dpa stained by immunofluorescence for cardiomyocytes (α-Myh11/MF20 antibody; green) and cycling cells (α-PCNA antibody; magenta). Boxed regions are enlarged in (1-3). (**E**) Cardiomyocyte proliferation indices at 7 dpa in DMSO- and NSC405020-treated hearts. Scale bar: in (B) 400 µm; in (D) 50 µm. Statistical significance in (A and C) was determined using one- way ANOVA with Tukey's multiple comparisons test, in (E) was determined using student's t- test. ns, not significant; *; $p<0.05$; **; $p<0.01$; ***, $p<0.001$.

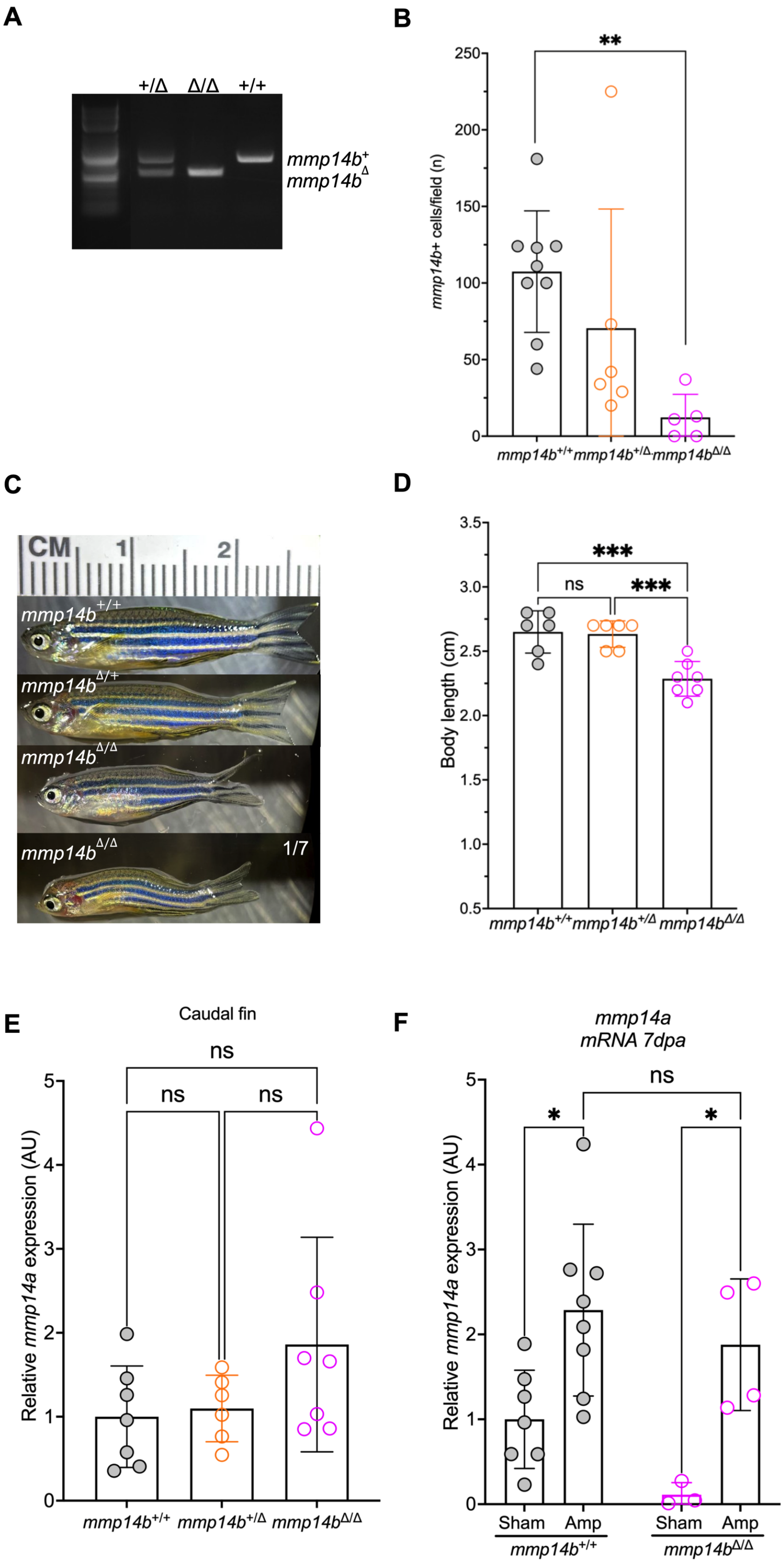

A
+/Δ
Δ/Δ
+/+
mmp14b+
mmp14bΔ
B
**
mmp14b+ cells/field (n)
250
200
150
100
50
0
mmp14b+/+
mmp14b+/Δ
mmp14bΔ/Δ
C
CM
1
2
mmp14b+/+
mmp14bΔ/+
mmp14bΔ/Δ
mmp14bΔ/Δ
1/7
D
***
ns
***
Body length (cm)
3.5
3.0
2.5
2.0
1.5
1.0
0.5
mmp14b+/+
mmp14b+/Δ
mmp14bΔ/Δ
E
Caudal fin
ns
ns
ns
Relative mmp14a expression (AU)
5
4
3
2
1
0
mmp14b+/+
mmp14b+/Δ
mmp14bΔ/Δ
F
mmp14a
mRNA 7dpa
ns
*
*
Relative mmp14a expression (AU)
5
4
3
2
1
0
Sham
Amp
Sham
Amp
mmp14b+/+
mmp14bΔ/Δ

Fig. S3. Genetic inactivation of Mmp14b function in zebrafish. (**A**) PCR genotyping of a homozygous *mmp14b*$^{\Delta/\Delta}$ mutant. (**B**) The number of *mmp14b*-positive cells in the heart is reduced in *mmp14b*$^{+/\Delta}$ and *mmp14b*$^{\Delta/\Delta}$ compared to wild type *mmp14b*$^{+/+}$ hearts at 7 dpa. (**C**) Representative images of *mmp14b*$^{+/+}$, *mmp14b*$^{+/\Delta}$, and *mmp14b*$^{\Delta/\Delta}$ zebrafish at 3 months age. The majority of *mmp14b*$^{\Delta/\Delta}$ zebrafish are phenotypically normal, albeit shorter. Rarely (1/7), *mmp14b*$^{\Delta/\Delta}$ zebrafish exhibit an evident skeletal phenotype (bottom panel). (**D**) The total body length of *mmp14b*$^{\Delta/\Delta}$ zebrafish is significantly shorter than *mmp14b*$^{+/+}$ and *mmp14b*$^{+/\Delta}$ zebrafish at 3 months. (**E**) Relative expression of *mmp14a* in *mmp14b*$^{+/+}$, *mmp14b*$^{+/\Delta}$ and *mmp14b*$^{\Delta/\Delta}$ zebrafish caudal fin without injury as measured by RT-qPCR. (**F**) Relative expression of *mmp14a* in *mmp14b*$^{+/+}$ and *mmp14b*$^{\Delta/\Delta}$ zebrafish hearts without injury (Sham) and injured (Amp) at 7 dpa as measured by RT-qPCR. Statistical significance was determined using one-way ANOVA with Tukey's multiple comparisons test. ns, not significant; *; $p<0.05$; **; $p<0.01$; ***, $p<0.001$.

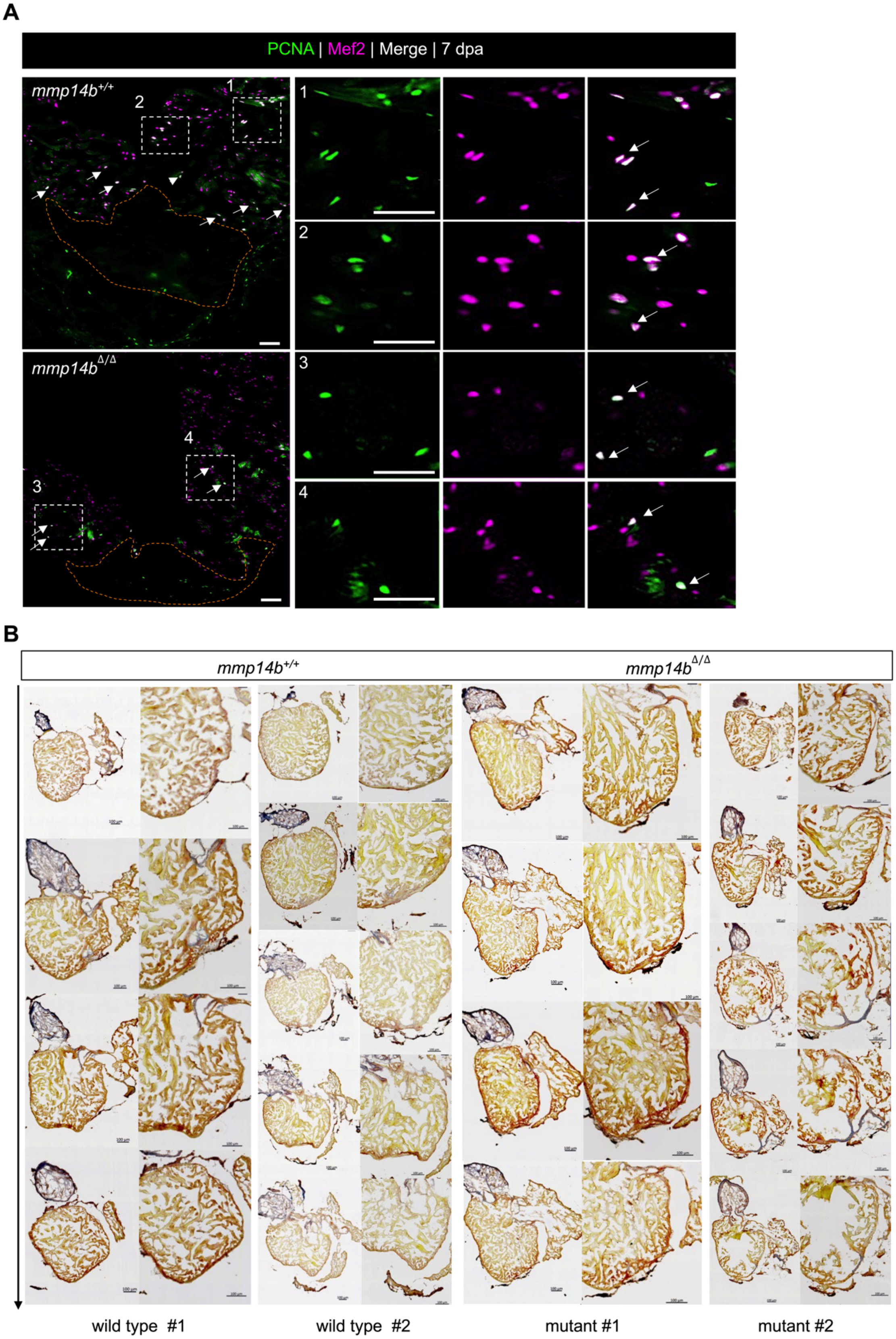

A
PCNA | Mef2 | Merge | 7 dpa
mmp14b+/+
mmp14bΔ/Δ
1
2
3
4
B
mmp14b+/+
mmp14bΔ/Δ
wild type #1
wild type #2
mutant #1
mutant #2

Fig. S4. Genetic inactivation of *mmp14b* inhibits cardiomyocyte proliferation and scar resolution in adult zebrafish. (**A**) Heart sections of *mmp14b*$^{+/+}$ and *mmp14b*$^{\Delta/\Delta}$ hearts 7 dpa stained for cardiomyocyte nuclei (α-Mef2 antibody; magenta) and cycling cells (α-PCNA antibody; green). Boxed regions are enlarged in (1-4). Arrows highlight PCNA/Mef2 double-positive cardiomyocytes. Scale bars, 50 µm. (**B**) Frontal sections of adult *mmp14b*$^{+/+}$ and *mmp14b*$^{\Delta/\Delta}$ zebrafish hearts collected at 30 dpa and stained with AFOG to detect muscle (brown), fibrin (red), and collagen (blue). Two wild type *mmp14b*$^{+/+}$ and two mutant *mmp14b*$^{\Delta/\Delta}$ hearts with 4-5 sections per heart shown. Higher magnification images are shown in the right columns for each individual. The *mmp14b*$^{+/+}$ and *mmp14b*$^{\Delta/\Delta}$ sections shown in Fig. 2E are distinct sections belonging to the same series as wild type #1 and mutant #1, respectively.

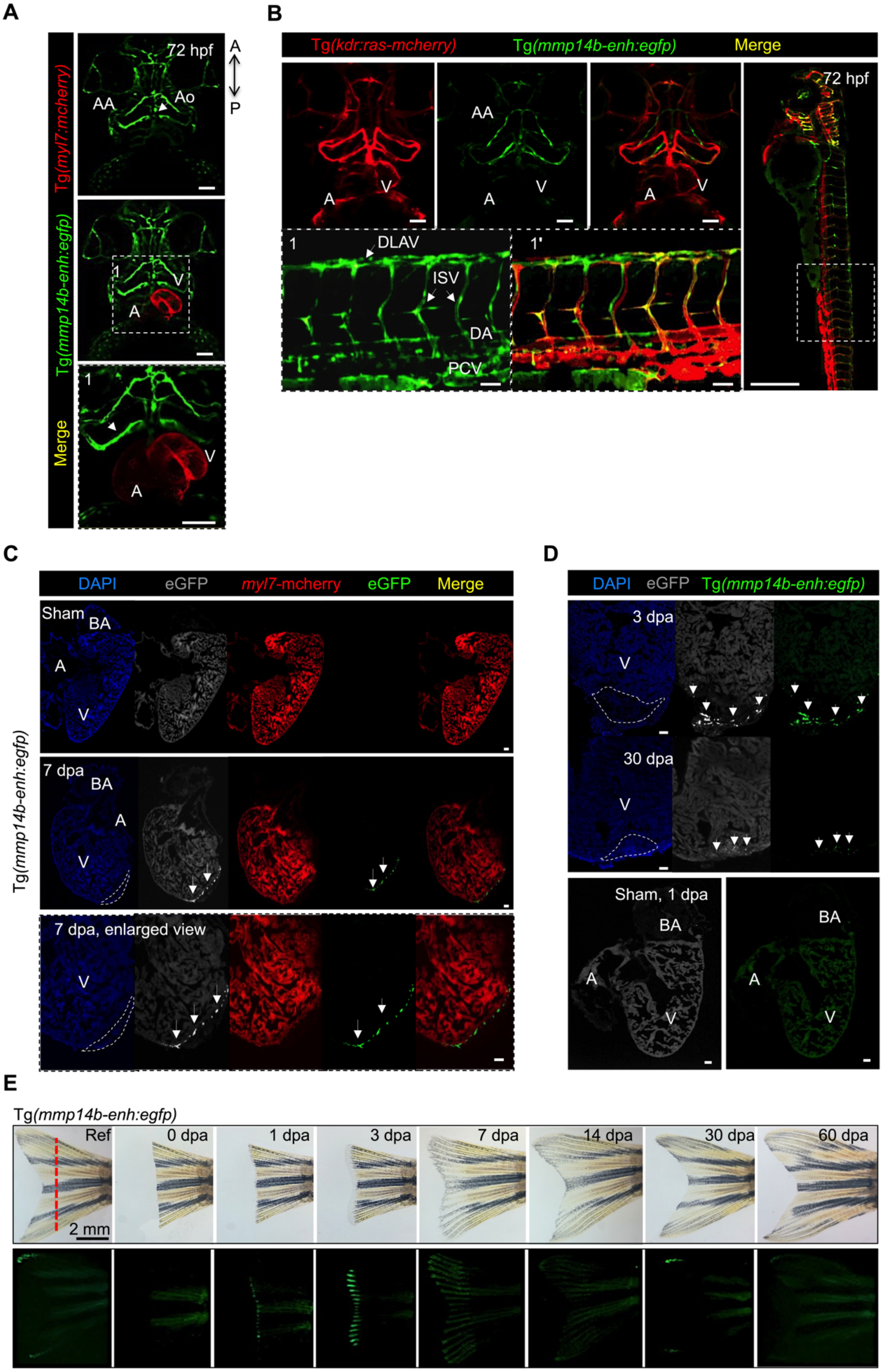
A
B
C
D
E
Tg(myl7:mcherry)
Tg(mmp14b-enh:egfp)
Merge
72 hpf
A
P
AA
Ao
V
A
1
Tg(kdr:ras-mcherry)
Tg(mmp14b-enh:egfp)
Merge
DLAV
ISV
DA
PCV
1'
DAPI
eGFP
myl7-mcherry
eGFP
Merge
Sham
BA
7 dpa
7 dpa, enlarged view
Tg(mmp14b-enh:egfp)
3 dpa
30 dpa
Sham, 1 dpa
Ref
0 dpa
1 dpa
3 dpa
7 dpa
14 dpa
30 dpa
60 dpa
2 mm

Fig. S5. ***mmp14b-enh*** **is activated in endothelial cells during development and in response to injury.** (**A** and **B**) Representative fluorescent images of zebrafish embryos at 72 hpf show endothelial-specific eGFP expression in Tg(*mmp14-enh:egfp*) zebrafish. (**A**) Tg(*myl7:mCherry*) expression marks cardiomyocytes in red. Merged images are shown in the lower panels, and the boxed region is enlarged in (1). Note the absence of expression in the endocardium. (**B**) Tg(*kdrl:ras-mCherry*) expression marks the vascular endothelium. A stable *mmp14b-enh:egfp* transgenic zebrafish line demonstrates endothelial-specific expression. Whole-mount views of the zebrafish embryo. The boxed region is enlarged and rotated in (**1, 1′**). (**C** and **D**) Representative fluorescent images frontal sections of adult Tg(*mmp14b-enh:egfp*) hearts at 7dpa (**c**) and at 3 and 30 dpa (**D**), showing eGFP (green), cardiomyocytes (mCherry, red), and nuclei (DAPI, blue). No detectable eGFP fluorescence was observed in sham-operated hearts. Minimal eGFP activity was observed at 30 dpa (**D**). Dashed lines mark the approximate injured/regenerated area. Arrows mark eGFP+ cells at the injured/regenerated area. (**E**) Representative bright field (upper) and fluorescent (lower) images of caudal fin regeneration in Tg(*mmp14b-enh:egfp*) zebrafish from 0-60 dpa. The dashed red line approximates the amputation plane. A, atrium; AA, aortic arches; Ao; aorta; CV, cardinal vein; DA, dorsal aorta; DLAV; dorsal longitudinal anastomotic vessel; ISV, intersegmental vessels; PCV, posterior cardinal vein; V, ventricle. Scale bars, 50 μm, except where indicated.

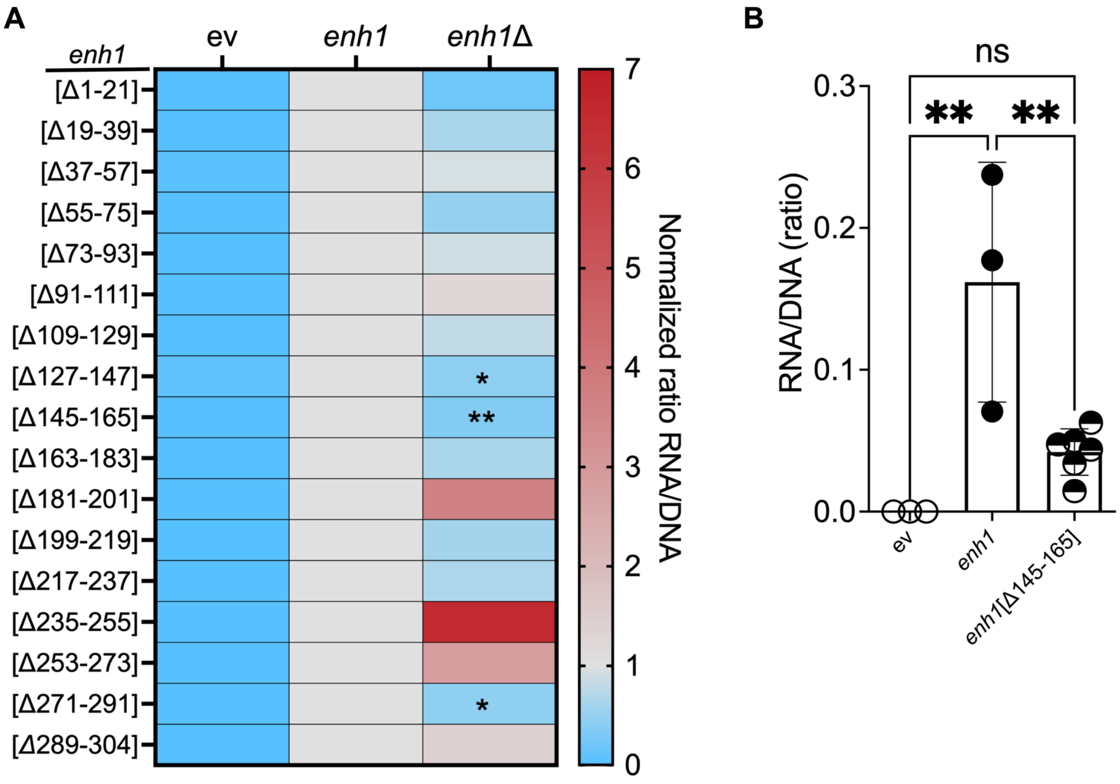

Fig. S6. Unbiased screen for *cis*-acting elements in *mmp14b-enh1* identifies a 21-bp region encompassing a functional TEAD binding site required for enhancer function *in vivo*. (**A**) Heat map depicting relative expression of reporter mRNA from each of 17 overlapping, 21-bp *mmp14b-enh1* deletions. Three deletions (Δ127-147, Δ145-165, and Δ271- 291) displayed significant ($p<0.05$) reduction in activity in transient transgenic analyses when compared to full length *mmp14b-enh1* (*enh1*, positive control). The empty *e1b-egfp(tag)* vector (ev) negative control is also shown. In particular, *mmp14b-enh1*[Δ145-165] exhibited the largest and most significant reduction in expression in zebrafish larvae at 72 hpf. (**B**) RT-qPCR analysis for reporter mRNA expression directed by the empty vector (ev) transgene plasmid, *mmp14b-enh1* (*enh1*), and *mmp14b-enh1*[Δ145-165] showed significantly decreased expression of *enh1*[Δ145-165] compared to *enh1*. Statistical significance was determined using one-way ANOVA with Tukey's multiple comparisons test. ns, not significant; *, $p<0.05$; **, $p<0.01$.

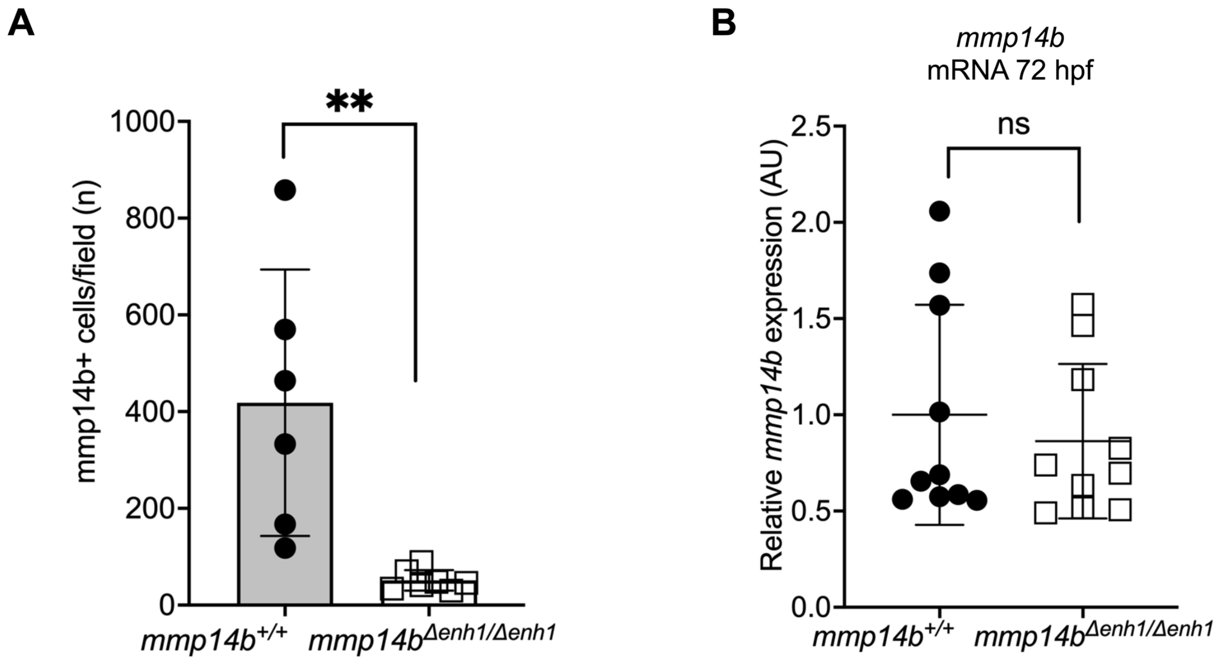

Fig. S7. *mmp14b* expression during development and post-injury in *mmp14b*$^{\Delta enh1/\Delta enh1}$ mutant and wild type zebrafish. (**A**) Quantification of *mmp14b*-positive cells in *mmp14b*$^{+/+}$ and *mmp14b*$^{\Delta enh1/1\Delta enh1}$ adult zebrafish hearts at 7 dpa. (**B**) Relative expression of *mmp14b* transcripts in *mmp14b*$^{+/+}$ and *mmp14b*$^{\Delta enh1/1\Delta enh1}$ zebrafish larvae at 72 hpf. Statistical significance was determined using student's t-test. ns, not significant; **, p<0.01.

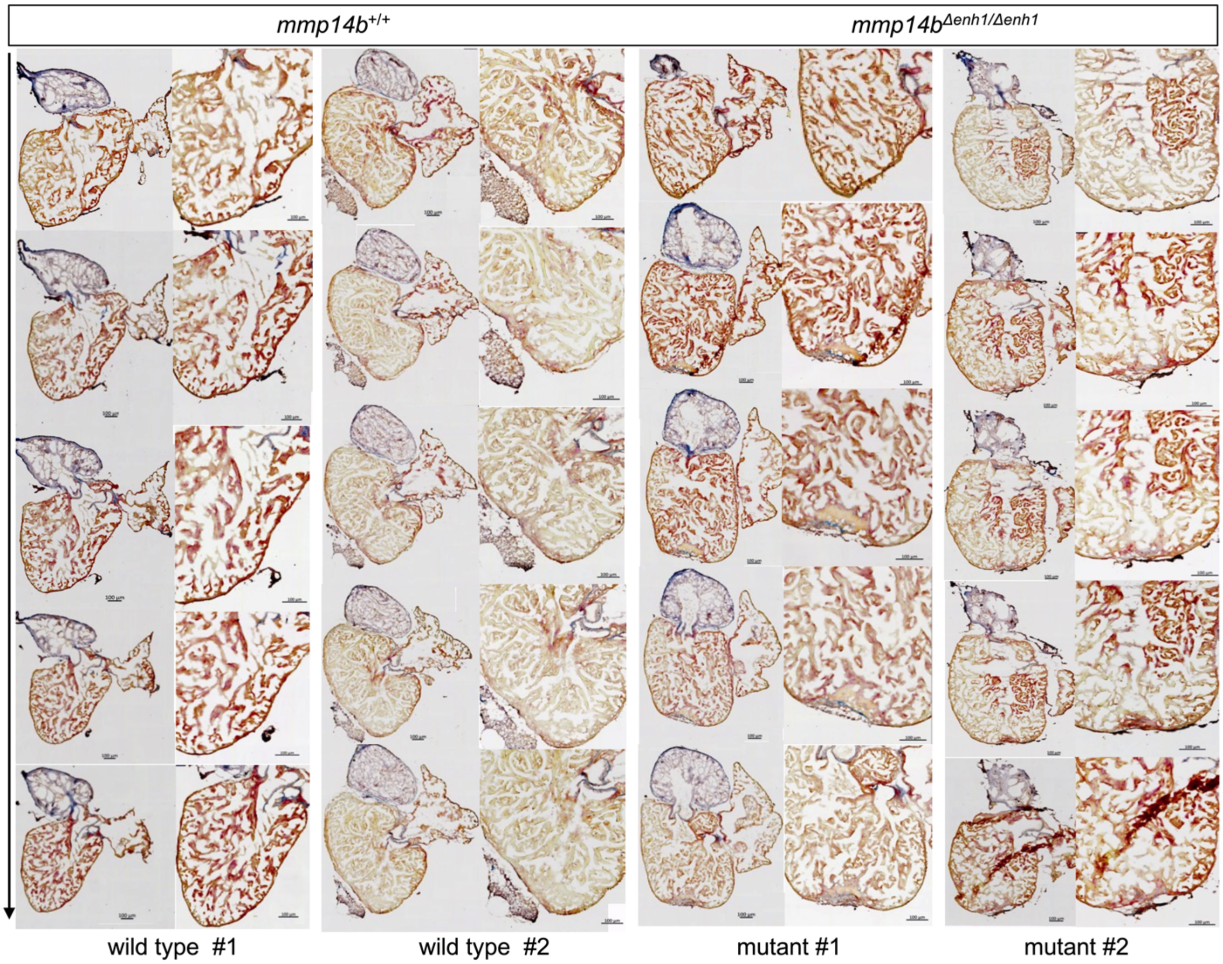

Fig. S8. Scar resolution in *mmp14b*$^{\Delta enh1/\Delta enh1}$ mutant and wild type zebrafish. Frontal sections of adult *mmp14b*$^{+/+}$ and *mmp14b*$^{\Delta enh1/\Delta enh1}$ zebrafish hearts collected 30 dpa and stained with AFOG to detect muscle (brown), fibrin (red), and collagen (blue). Two wild type *mmp14b*$^{+/+}$ and two mutant *mmp14b*$^{\Delta enh1/\Delta enh1}$ with 5 sections per heart shown. Higher magnification images are shown in the right columns for each individual. The *mmp14b*$^{+/+}$ and *mmp14b*$^{\Delta enh1/1\Delta enh1}$ sections shown in Fig. 5G are distinct sections belonging to the same series as wild type #1 and mutant #1, respectively.

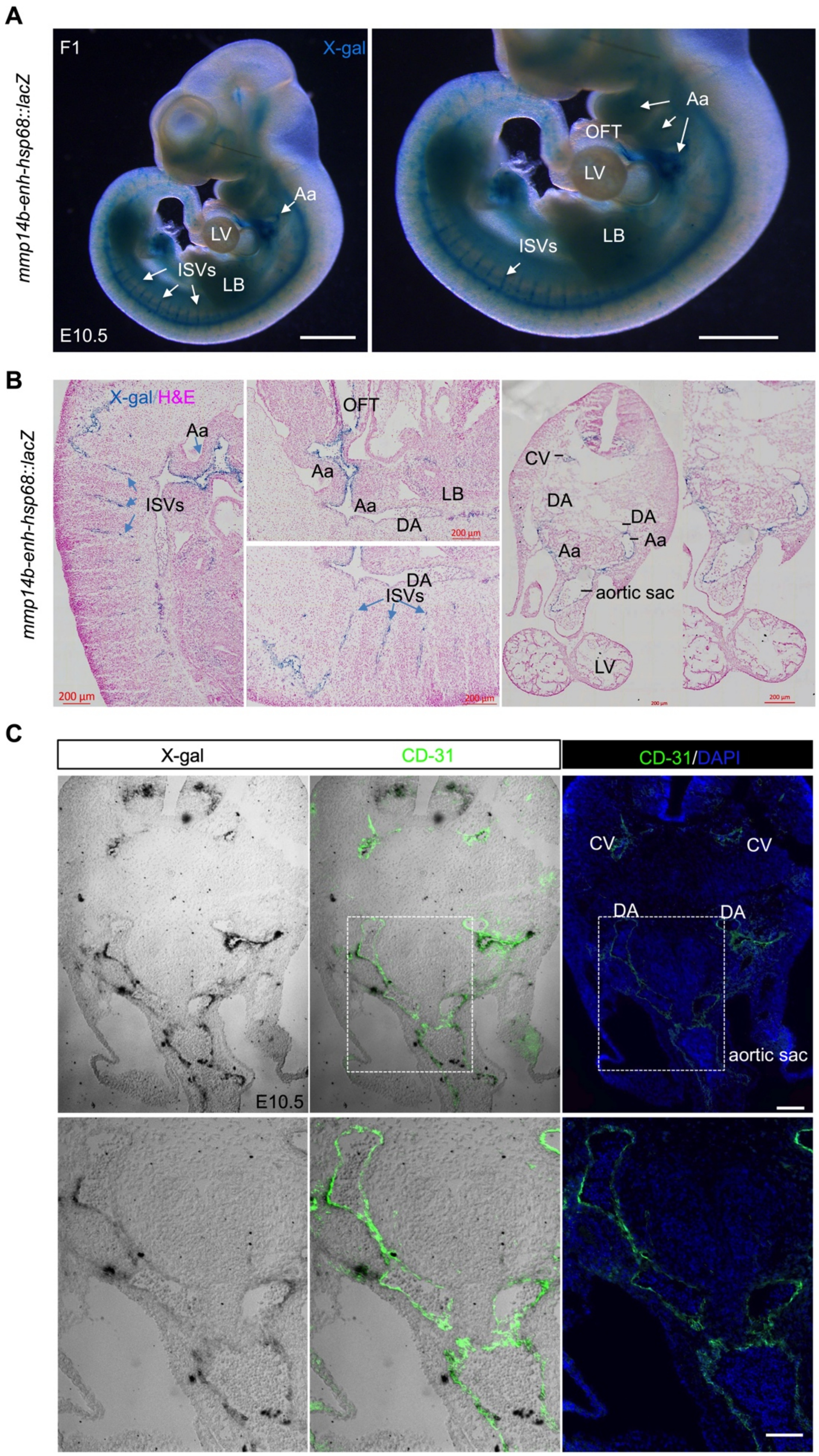
A
mmp14b-enh-hsp68::lacZ
F1
X-gal
Aa
LV
ISVs
LB
E10.5
Aa
OFT
LV
ISVs
LB
B
mmp14b-enh-hsp68::lacZ
X-gal/H&E
Aa
ISVs
200 μm
OFT
Aa
Aa
LB
DA
200 μm
DA
ISVs
200 μm
CV
DA
DA
Aa
Aa
aortic sac
LV
200 μm
C
X-gal
CD-31
CD-31/DAPI
CV
CV
DA
DA
aortic sac
E10.5

Fig. S9. Zebrafish *mmp14b-enh* is active in the developing vascular endothelium in mice. Representative images of whole-mount (**A**) and sagittal (**B**, left 3 panels) and transverse (**B**, right two panels and **C**) sections of E10.5 *mmp14b-enh-hsp68::lacZ* embryos stained with X-gal show endothelial-specific activity of zebrafish *mmp14b-enh* in mice. Note the absence of staining in the endocardium, as in the zebrafish. Sections in (B) have been counterstained with hematoxylin and eosin (H&E, pink). Sections in (C) have been co-immunostained for the endothelial cell marker CD31 (green); overlap with X-gal (middle) and DAPI (right) is shown. The dashed boxes in (C, upper row) are shown in magnified views in the lower panels. Note the extensive overlap of β-galactosidase activity (X-gal) and CD31. AA, aortic arches; cardinal vein; DA, dorsal aorta; DLAV; ISV, intersomitic vessels; LB, limb bud; LV, left ventricle; OFT, outflow tract. Scale bars: in (A), 1 mm; (B), 200 µm; (C), 100 µm.

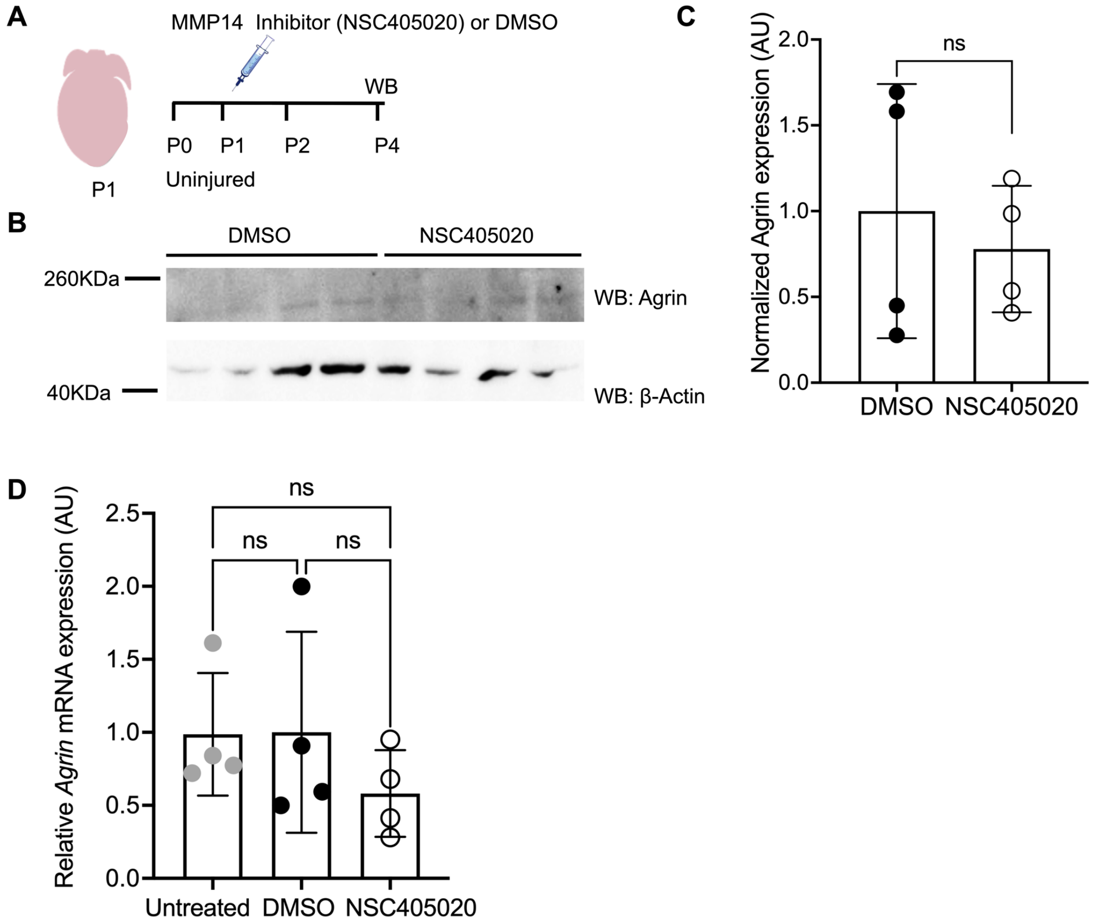

Fig. S10. **Basal expression of Agrin in uninjured hearts of neonatal mice treated with NSC405020.** (**A**) Schematic of the experimental design for MMP-14 inhibitor treatment in uninjured neonatal mice. Mice were treated with a single intraperitoneal injection of 20 µM NSC405020 or DMSO on P1, and hearts were collected on P4 for analysis by western blot (WB). (**B**) Western blots for Agrin and β-actin from total heart tissue. (**C**) Quantification of the 260kD isoform of Agrin protein expression (normalized to the expression of β-actin) in uninjured neonatal hearts treated with DMSO or NSC405020. (**D**) Relative expression of *Agrin* mRNA by qPCR in untreated, DMSO-treated, or NSC405020-treated P4 neonatal hearts. Statistical significance was determined in (**C**) using student's t-test. Statistical significance in (**D**) was determined using one-way ANOVA with Tukey's multiple comparisons test. ns, not significant.

Oligonucleotides	Forward Primer	Reverse Primer
**Cloning**		
mmp14b-enh	5’-ctccatatgggctcttttcttcctctgtttc-3’	5’-aaaacatttctaattgcaggtcaatgactactagtttc-3’
mmp14b-enhΔ1	5’-cttcataggacagaaccgcaggaaatagcccaggg-3’	5’-tttctctgttagtctctcttttctaaaatgacatc-3’
mmp14b-enh1[*mTEAD*]	5’-aagggggtggtagcttggagaatatagacaaagag-3’	5’-attctccaagctaccaccccccttcctcatcccactt-3’
**Genotyping**		
mmp14b-enh	5’-ggatgacatctgtcctcagttac-3’	5’-gcaaagatcttgctggttgttc-3’
mmp14b	5’-cttgcgaccttagttggct-3’	5’-agggacctctatgtggttaca-3’
		5’-caaaccagacatgtcaaatgtagta-3’
**Cloning-Barcoding**		
*mmp14b-enh1[*Δ1]	5’-ctaaaatgacatcacttttcagcctacaatactt-3’	5’-gtgatgtcattttagtcgagggcccatctggcctg-3’
mmp14b-enh1[Δ2]	5’-cttgcgaccttagttggct-3’	5’-agtattgtaggctgaagagagactaacagagaatc-3’
mmp14b-enh1[Δ3]	5’-tattctttatgcagaattgcatcattcacaccca-3’	5’-tctgcataaagaatagtgatgtcattttagaaaag-3’
mmp14b-enh1[Δ4]	5’-gcatcattcacacccaccgcgtttcctgcccgaa-3’	5’-gggtgtgaatgatgcagtattgtaggctgaaaagt-3’
mmp14b-enh1[Δ5]	5’-gcgtttcctgcccgaaaacatcaatcccattttc-3’	5’-tcgggcaggaaacgctctgcataaagaatagaaag-3’
mmp14b-enh1[Δ6]	5’-catcaatcccattttccccccatctctttgtcta-3’	5’-aaaatgggattgatggggtgtgaatgatgcaattc-3’
mmp14b-enh1[Δ7]	5’-cccatctctttgtctatattctccaacattccac-3’	5’-agacaaagagatgggtcgggcaggaaacgcggtgg-3’
mmp14b-enh1[Δ8]	5’-ttctccaacattccaccccccttcctcatcccac-3’	5’-tggaatgttggagaaaaaatgggattgatgttttc-3’
mmp14b-enh1[Δ9]	5’-ccttcctcatcccacttccttcctcaaactctgt-3’	5’-gtgggatgaggaaggagacaaagagatgggggggaa-3’

mmp14b-enh1[Δ10]	5’-cttcctcaaactctgtcactacatcaccaaccgt-3’	5’-cagagtttgaggaagtggaatgttggagaatatag-3’
mmp14b-enh1[Δ11]	5’-ctacatcaccaaccgttggcatcatcgcacatct-3’	5’-cggttggtgatgtaggtgggatgaggaagggggtg-3’
mmp14b-enh1[Δ12]	5’-gcatcatcgcacatctgctctgaagccatgtggt-3’	5’-gatgtgcgatgatgccagagtttgaggaaggaagt-3’
mmp14b-enh1[Δ13]	5’-tctgaagccatgtggtcaaaaaataaaaaaatcc-3’	5’-ccacatggcttcagacggttggtgatgtagtgaca-3’
mmp14b-enh1[Δ14]	5’-aaaaataaaaaaatccctgggctatttcctgcgg-3’	5’-gattttttatttttgatgtgcgatgatgccaacg-3’
mmp14b-enh1[Δ15]	5’-gggctatttcctgcggttctgtcctatgaaggat-3’	5’-cgcaggaaatagcccccacatggcttcagagcaga-3’
mmp14b-enh1[Δ16]	5’-ctgtcctatgaaggatctctcgactctagagggt-3’	5’-tccttcataggacaggatttttttatttttgacc-3’
mmp14b-enh1[Δ17]	5’-gatctctcgactctagagggtatataatggatcc-3’	5’-tagagtcgagagatccgcaggaaatagcccaggga-3’
**EMSA**		
TEAD wt	5’-ggggtggaatgttggaga-3’	5’-gggtctccaacattccacc-3’
TEAD muta1	5’-ggggtggtaggttggaga-3’	5’-gggtctccaacctaccacc-3’
TEAD muta2	5’-ggggtggtagcttggaga-3’	5’-gggtctccaagctaccacc-3’
**qPCR**		
ef1a	5’-cttctcaggctgactgtgc-3’	5’-ccgctagcattaccctcc-3’
mmp14b	5’-aatggcaaggcgttccagacaaca-3’	5’-ctctcacgttcccggtctcggtca-3’

Table S1. Oligonucleotides used in these studies.

REAGENT or RESOURCE	SOURCE	IDENTIFIER
**Antibodies**		
Mouse monoclonal MF20	Developmental Studies Hybridoma Bank	DSHB Cat# MF20; RRID: AB_2147781
Mouse monoclonal [PC10] to PCNA	Abcam	Cat# ab201672; RRID: N/A
Rabbit polyclonal anti-Mef2	Santa Cruz Biotech	Cat# sc-313; RRID: AB_631920
Chicken polyclonal anti-GFP	Abcam	Cat# ab13970; RRID: AB_300798
anti-beta Galactosidase	Abcam	Cat#ab9361; RRID: AB_307210
Rabbit polyclonal anti-CD31	Abcam	Cat#ab28364; RRID: AB_726362
Rabbit polyclonal to Agrin (WB)	Abcam	Cat#ab85174; RRID: AB_1860988
Anti-beta-actin clone AC-74 (WB)	Sigma	Cat#A228; RRID: N/A
Goat anti-Rabbit IgG (H+L) Cross-Absorbed Secondary Antibody, Alexa Fluor 488	Thermo Fisher Scientific	Cat# A-11008; RRID: AB_14165
Donkey Anti-Rabbit IgG (H+L) Secondary Alexa Fluor® 647 AffiniPure	Jackson, ImmunoResearch	Cat#711_605_152; RRID: AB_2492288
Goat anti-Mouse IgG (H+L) Cross-Absorbed Secondary Antibody, Alexa Fluor 488	Thermo Fisher Scientific	Cat# A-11029; RRID: AB_2534088
Goat anti-Mouse IgG (H+L) Cross-Absorbed Secondary Antibody, Alexa Fluor 594	Thermo Fisher Scientific	Cat# A-11005; RRID: AB_2534073
Goat anti-chicken IgG (H+L) Cross-Absorbed Secondary Antibody, Alexa Fluor 594	Thermo Fisher Scientific	Cat# A-11042; RRID: AB_2534099
Anti-rabbit IgG, HRP-linked Antibody (WB)	Cell Signaling Technology	Cat# 7074S; RRID: AB_2099233
EasyBlot anti Mouse IgG (HRP)(WB)	GeneTex	Cat# GTX221667-01; RRID: AB_10728926
**RNA-scope assays**		
RNAscope® Multiplex Fluorescent Reagent Kit v2 - RNAscope® Multiplex Fluorescent Reagent Kit v2. RNA in situ hybridization (ISH)	ACD	Cat#323100
RNAscope® Probe - Dr-*mmp14b*-C1-Danio rerio matrix metallopeptidase 14b	ACD	Cat#1061661-C1

RNAscope® Negative Control Probe - DapB - Bacillus subtilis strain SMY methylglyoxal synthase (mgsA) gene; dihydrodipicolinate reductase (dapB) gene,	ACD	Cat#310043
RNAscope® Probe - Dr-*fli1a*-C2	ACD	Cat#573351-C2
RNAscope® Probe - egfp-C3	ACD	Cat#400281-C3
TSA PLUS CYANINE	Akoya	Cat# NEL745001KT
TSA PLUS CYANINE 3	Akoya	Cat#NEL744001KT
TSA PLUS FLUORESCEIN	Akoya	Cat#NEL741001KT
VECTASHIELD Antifade Mounting Medium	Vector Laboratories	Cat# H-1000; RRID: AB_2336789
ProLong Gold Antifade Mountant	Life Technologies	Cat#P36930; RRID:SCR_015961

Table S2. Reagents and antibodies used in these studies.

Data S1. (separate file). Coordinates and neighboring genes of accessible chromatin regions identified in ATAC-seq from endothelial cells from injured versus uninjured zebrafish hearts.
